# Differentiation is accompanied by a progressive loss in transcriptional memory

**DOI:** 10.1186/s12915-024-01846-9

**Published:** 2024-03-12

**Authors:** Camille Fourneaux, Laëtitia Racine, Catherine Koering, Sébastien Dussurgey, Elodie Vallin, Alice Moussy, Romuald Parmentier, Fanny Brunard, Daniel Stockholm, Laurent Modolo, Franck Picard, Olivier Gandrillon, Andras Paldi, Sandrine Gonin-Giraud

**Affiliations:** 1 Laboratoire de Biologie et Modélisation de la Cellule, Ecole Normale Supérieure de Lyon, CNRS, UMR5239, Université Claude Bernard Lyon 1, Lyon, France; 2 Ecole Pratique des Hautes Etudes, PSL Research University, Sorbonne Université, INSERM, CRSA, Paris, 75012 France; 3 Plateforme AniRA-Cytométrie, Université Claude Bernard Lyon 1, CNRS UAR3444, Inserm US8, ENS de Lyon, SFR Biosciences, Lyon, F-69007 France; 4 Inria Center, Grenoble Rhone-Alpes, Equipe Dracula, Villeurbanne, F69100 France

**Keywords:** Cell differentiation, Gene expression variability, Transcriptome, Sister cells, Transcriptional memory

## Abstract

**Background:**

Cell differentiation requires the integration of two opposite processes, a stabilizing cellular memory, especially at the transcriptional scale, and a burst of gene expression variability which follows the differentiation induction. Therefore, the actual capacity of a cell to undergo phenotypic change during a differentiation process relies upon a modification in this balance which favors change-inducing gene expression variability. However, there are no experimental data providing insight on how fast the transcriptomes of identical cells would diverge on the scale of the very first two cell divisions during the differentiation process.

**Results:**

In order to quantitatively address this question, we developed different experimental methods to recover the transcriptomes of related cells, after one and two divisions, while preserving the information about their lineage at the scale of a single cell division. We analyzed the transcriptomes of related cells from two differentiation biological systems (human CD34+ cells and T2EC chicken primary erythrocytic progenitors) using two different single-cell transcriptomics technologies (scRT-qPCR and scRNA-seq).

**Conclusions:**

We identified that the gene transcription profiles of differentiating sister cells are more similar to each other than to those of non-related cells of the same type, sharing the same environment and undergoing similar biological processes. More importantly, we observed greater discrepancies between differentiating sister cells than between self-renewing sister cells. Furthermore, a progressive increase in this divergence from first generation to second generation was observed when comparing differentiating cousin cells to self renewing cousin cells. Our results are in favor of a gradual erasure of transcriptional memory during the differentiation process.

**Supplementary Information:**

The online version contains supplementary material available at 10.1186/s12915-024-01846-9.

## Background

During cell division, the mother cell endures a period of transient instability-the mitosis-which is accompanied by dramatic cellular and epigenomic reorganizations [1]. The close to equal partitioning of the cellular content, together with active mechanisms, such as the conservation of gene transcription profiles after division by chromatin-related epigenetic mechanisms, or the long half-life of proteins ensure the overall phenotypic similarity of the sibling cells [2–4]. As a consequence, the resulting sister cells regain immediately after the division many of the structural and functional features of the maternal cell. The phenotypic stability of clonal cell lines is largely founded on this phenomenon frequently called “cellular memory.”

In the present work, we will focus on transcriptional memory defined as the closer proximity in term of mRNA content of related cells as compared to randomly paired cells. Such a memory is thought to be quite transient, happening over the course of a few cell divisions and is directly related to the maintenance of transient cell states. A small number of studies have addressed the question of the preservation of this transcriptional memory through division using different approaches ranging from microfluidics combined with scRNA-seq (single-cell RNA sequencing) [5], to time-lapse microscopy of reporter genes expression [6, 7], to a dedicated procedure called MemorySeq [8]. Most of those studies have been focused on self-renewing cells, such as mouse embryonic stem cells or melanoma cell line. In all cases, the authors concluded to the existence of a transcriptional memory defined by the heritability of gene expression levels in a gene-specific manner, extending up to two or more generations. This transcriptional memory impacts subsets of genes called “memory genes,” the expression of which is uncorrelated in a population of cells but correlated in sister cells. Those genes are highly dependent on the cell system used for the investigation. Beyond their actual function, the fact that related cells harbor correlated expression for those genes is a read-out for this transcriptional memory and demonstrates the existence of a constraint imposed to the cells gene expression profile at division.

On the other hand, all cellular processes are subject to stochastic molecular fluctuations, which favor the decorrelation of sister cells phenotypes and increase transcriptional heterogeneity in a clonal population of siblings. For example, relaxation experiments demonstrated on various cell systems that after 2 weeks of culture under stable conditions, the expression level of specific genes in a selected homogeneous cell clone becomes as heterogeneous as it was in the original population the founder cell derived from [9]. Moreover, the capacity of a cell clone to reconstitute the heterogeneity of the original population over time has been observed in many instances in normal or pathological cell types [4, 8, 10].

**Fig. 1:**
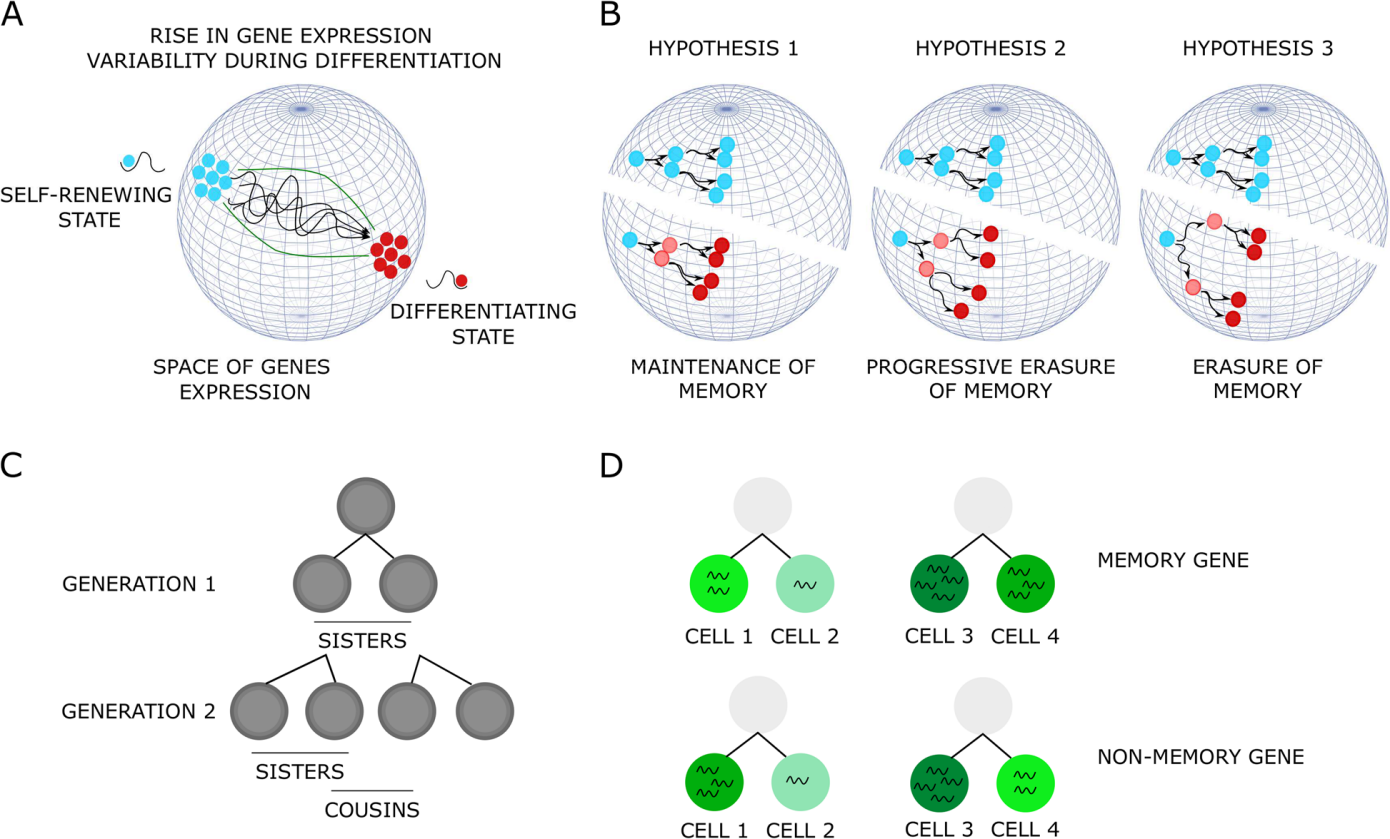
Representation of the concepts used in this study. (**A**) Schematic representation of a dynamic differentiation process. If one assumes that cells are dots moving in a gene expression space (sphere), then one can represent cells in a 3D (i.e. 3 genes) space. Self-renewing cells (blue cells) display some micro-heterogeneity, as well as differentiating cells (red cells). The differentiation process is accompanied by an increase in cell-to-cell variability (i.e. macro-heterogeneity) that allows the cells to escape locally attractive state and attain a new differentiated state [23–25]. (**B**) Three possible hypotheses on transcriptional memory behavior during a differentiation process (see text for details). (**C**) The type of genealogical information that was made available by dedicated methods in this paper. (**D**) Schematic description of a memory gene (upper) and a non-memory gene (lower). In the case of a memory gene, the geometric distance between the sister-cells 1 and 2 is of 2 minus 1, that is 1. The distance between the sister-cells 3 and 4 is of 4 minus 3, that is also 1. The mean distance for the memory gene between all sister-cells is therefore of 1, whereas the mean distance for the non-memory gene between all sister-cells is of 2 in this example

During the process of differentiation, this whole delicate balance between the two opposing forces of the stabilizing cellular memory and change-inducing gene expression fluctuations has to be somehow revisited. Indeed, differentiating cells undergo substantial morphological and functional changes. Although differentiation usually takes place over several cell cycles, there is a critical transition period characterized by stochastic gene expression and rapid morphological fluctuations. A large range of experimental studies have indeed demonstrated, that the first step in cell differentiation is the rapid and transient increase of the variability in gene expression in response to the stimuli inducing the differentiation, both in vitro [11–20] and in vivo [21, 22] (Fig. 1A).

An important unresolved question is therefore to understand how the dynamic stability and the capacity of differentiation are integrated into a single process. In the present study, we aimed to investigate the dynamic balance of stability/instability in dividing cells that undergo the first steps of differentiation. To do this, we measured the resemblance of the sister cells by comparing their transcriptomes.

We formulated 3 hypotheses on the possible evolution of transcriptional memory upon differentiation induction (Fig. 1B). To illustrate those hypotheses, cells in a self-renewing state are positioned in a gene expression space. Assuming the existence of transcriptional memory in our self-renewing cells after mitosis, like in other cell models, sister cells start in roughly at the same position in that space (blue family tree). Then, upon differentiation induction (red family tree), we can postulate the following three hypotheses:

- The maintenance of memory hypothesis: the transcriptional memory overrules the expression variability resulting in related cells following roughly the same path in the gene expression space toward the differentiated state (hypothesis 1), or
- The progressive erasure of memory hypothesis: the memory is gradually erased, translated in our projection to differentiating sister cells starting to follow roughly the same path and progressively bifurcating from each other, and even more after one more cell division (hypothesis 2), or
- The instantaneous erasure of memory hypothesis: the variability of gene expression pushes the balance and takes over the transcriptional memory, leading each differentiating sister cell to follow a completely different path from the beginning of the differentiation process (hypothesis 3).

In order to distinguish between the different scenarios cited above, it is necessary to quantitatively evaluate, at the single-cell level, the similarity of the gene expression profiles of sister cells shortly after the division under self-renewing versus under differentiation-promoting conditions (Fig. 1C, D). Microfluidic approaches are the state-of-the-art methods to address those types of questions. For instance, Kimmerling and co-authors employed a microfluidic chip that allowed them to capture individual cells in dynamic traps, enabling the cells to divide and subsequently trapping the daughter cells in new traps. They compared the transcriptome proximity of two cell types (a mouse lymphocytic leukemia cell line L1210 and primary CD8+ activated T cells) and concluded that related cells present closer transcriptomics profiles than unrelated cells [5]. Another microfluidic approach, coupling imaging and single-cell tracking, called TraqSeq and developed by Wehling and co-authors was used to question fate-divergence during hematopoietic stem cells (HSC) asymmetric divisions [26]. However, it is not always possible to access complex microfluidic devices. Another type of approach that can be used in this context consists of genetically tagging the cells in order to generate genetic barcodes which will be passed along progenies [27]. However, those methods are sometimes out of reach for some primary cells which have quite a short lifespan and can be extremely hard to modify genetically.

Therefore, we developed two strategies to isolate cells while preserving their precise lineage information after one (generation 1) and two (generation 2) divisions, a manual one and a FACS-based one. Then, in order to assess the genericity and robustness of our findings, we compared two different cell differentiation models (human CD34+ cells and T2EC-TGF$\alpha$, TGF$\beta$ induced erythrocytic cells-chicken primary erythrocytic progenitors) and for the T2EC model two cellular states: self-renewing and differentiating. We used two different single-cell transcriptomics methods: a highly sensitive targeted quantification method, scRT-qPCR (single-cell reverse transcription-quantitative polymerase chain reaction), and a whole-transcriptome approach, scRNA-seq.

We obtained qualitatively very similar results using the two cell types and the two single-cell measurement technologies. First, after one cell division (generation 1) in both models, and in both states for the T2EC model, we detected a transcriptional memory demonstrated by the sister cells displaying more transcriptomic similarity between each other than two randomly selected cells. Second, using the T2EC model, which allows to compare sister cells induce to differentiate to sister cells in self-renewing state, we also observed that this transcriptome similarity decreased during the differentiation process as compared to the self-renewing cells. Interestingly, this effect was even more pronounced one division later (generation 2), when interrogating cousin cells. Altogether, our results point toward a gradual loss of transcriptional memory during the differentiation sequence.

## Methods

### Cell culture

Human hematopoietic CD34+ cells were purified from umbilical cord blood from three anonymous healthy donors. First, mononuclear cells were isolated by density centrifugation using Ficoll (Biocoll, Merck Millipore). CD34+ cells were then enriched by immunomagnetic beads using the AutoMACSpro (Miltenyi Biotec). Cells were frozen in 90% fetal bovine serum (Eurobio) 10% dimethyl sulfoxide (Sigma) and stored in liquid nitrogen. After thawing, cells were grown in prestimulation medium made of X-vivo (Lonza) supplemented with penicillin/streptomycin (respectively 100 U/mL and 100 *µ*g/mL-Gibco, Thermo Fisher Scientific), 50 ng/ml h-FLT3-ligand (FMS-like tyrosine kinase 3), 25 ng/ml h-SCF (stem cell factor), 25 ng/ml h-TPO (human Thrombopoietin), and 10 ng/ml h-IL3 (Miltenyi) final concentration as previously described [15]. Cells were cultured in a 96-well plate at 185,000 cells/mL during 24 h in a humidified 5% CO_2_ incubator at 37 ^∘^C before proceeding to mother cell isolation.

Cell population mortality was assessed by counting dead and living cells from the different time points and conditions after Trypan blue staining and using a Malassez cell.

T2EC cells were extracted from 19-day-old SPAFAS white leghorn chicken’s embryos’ bone marrow (INRA, Tours, France). Cells were grown in LM1 medium ($\alpha$-MEM, 10% fetal bovine serum (FBS), 1 mM HEPES, 100 nM $\beta$- mercaptoethanol, 100 U/ mL penicillin and streptomycin, 5 ng/mL TGF-$\alpha$, 1 ng/mL TGF-$\beta$, and 1 mM dexamethasone) as previously described [28]. T2EC cell differentiation was induced by removing LM1 medium and placing the cells into DM17 medium ($\alpha$-MEM, 10% fetal bovine serum (FBS), 1 mM Hepes, 100 nM $\beta$-mercaptoethanol, 100 U/mL penicillin and streptomycin, 10 ng/mL insulin, and 5% anemic chicken serum [29]).

### Manual strategy for CD34+ sister cell isolation

Mother cells were isolated using a SmartAliquotor (iBioChips). It consists of a polydimethylsiloxane chip divided into 100 wells (2 *µ*L per well, 1.8 mm of diameter) connected by microchannels to an insertion hole in the center. This system allows to physically isolate cells while sharing the same medium. Two hundred microliters of cell suspension at 1000 cells/mL were injected in the chip through the injection plug, and cells were randomly divided into the wells. Air bubbles were removed with sterile tips. Using a standard confocal microscope, wells containing lonely cells were listed. Twenty milliliters of prestimulation medium (see the “Cell culture” section for composition) were added to avoid evaporation, and cells were incubated at 37 ^∘^C in a humidified 5% atmosphere during 24 to 48 h. Listed wells were regularly checked with standard confocal microscope to identify cell division. Sister cells were manually collected under biological safety cabinet to keep sterile conditions and avoid impurities to fall in the culture dish. A micromanipulator connected to a flexible microfluidic capillary filled with PBS and ending in a 2-*µ*L glass microcapillary was used. Individual collected cells were immediately inserted into 5 *µ*L of lysis buffer (Triton 4% (Sigma), RNaseOUT Recombinant Ribonuclease Inhibitor 0.4 U/*µ*L (Thermo Fisher Scientific), nuclease-free water (Thermo Fisher Scientific), and spikes 1 and 4 (Fluidigm C1 Standard RNA Assays)) and kept on dry ice to preserve RNA. Particular attention has been given to preserve cells integrity. Samples were kept at − 20 ^∘^C until further scRT-qPCR analysis.

### FACS-oriented strategy for T2EC sister cell isolation

Mother cells were stained using CFSE (Cell Trace CFSE (carboxyfluorescein diacetate succinimidyl ester) Cell Proliferation kit (Thermo Fisher Scientific)); 5 × 10^5^ cells were placed in a 60-mm plate in 5 mL of culture medium mixed with 5 *µ*L of CFSE at 5 mM (final concentration 5$\mu$M) and incubated at 37 ^∘^C for 30 min. Cells were then centrifuged at 20 ^∘^C, 1500 rpm for 5min. Medium was discarded, and cells were resuspended in 5 mL fresh medium. CFSE-stained mother cells were then isolated using the CellenONE X1 (CELLENION) at CELLENION core facility (Lyon, France). A gating based only on morphological criteria (diameter, elongation, and circularity) was performed to select single living cells. Selected single cells were sorted in a 384-well plate containing 10 *µ*L of culture medium (either self-renewing medium LM1 or differentiation-inducing medium DM17). The plate was then kept in an incubator under 5% CO_2_, 37 ^∘^C for at least 20 h to allow one cell division. Each well of the 384-well plate was manually checked under a regular inverted microscope to identify cells that had undergone one cell division (presence of cell doublets). Each doublet was then harvested and placed in a FACS polypropylene tube containing 80 *µ*L of warm culture medium. Tubes containing cell doublets were kept at room temperature throughout the sorting process and were briefly vortex immediately before loading into the sorter. Prior settings consisted in analyzing the CFSE positive population, the CFSE negative population, and the culture medium. No fluorescent signal was ever detected in medium or in negative population (Additional file 1: Fig. S1A–B self-renewing medium and C–D differentiation medium) indicating that only cells of interest ever gave CFSE positive signal. Cells were sorted at 20 PSI (pounds per square inch) through a 100-*µ*m nozzle on a FACS AriaII (BD). Gating was performed on FSC-A/SSC-A to capture live cells, SSC-H /SSC-A to capture single cells, and CFSE positive cells with yield, purity, and phase mask of 32, 0, and 0 respectively. Those parameters were chosen because of the cell density being very low (2 cells per tube); the probability of the two cells being in two consecutive drops was extremely low. Furthermore, those parameters are very conservatives, and thus probability of the cell not being sorted is also very low. Cells were isolated in 4 *µ*L of lysis buffer in PCR tubes containing cell barcode primers. Tubes were frozen in dry ice directly after sorting to prevent any degradation of the samples.

### FACS-oriented strategy for T2EC cousin cell isolation

#### Fluorescent barcoding for lineage tracing

On the first day, 1 × 10^6^ mother cells were labeled with 0.5 *µ*M CTV (Cell Trace Violet Cell Proliferation kit (Thermo Fisher Scientific)) for 20 min at 37 ^∘^C in PBS; then, 5 mL of medium was added for 5 min to dilute the fluorescent molecules. The cells were centrifuged for 5 min at 1500 rpm at 20 ^∘^C, resuspended, and then separated into 6 tubes (2 × 10^5^ cells per tube) and resuspended in 1 mL per tube. Each sample was labeled with a different concentration of CFSE (3-point range of 5 *µ*M, 2.187 *µ*M, and 0.312 *µ*M) plus or minus CTY (10 *µ*M-Cell Trace Yellow Cell Proliferation kit (Thermo Fisher Scientific)) for 30 min at 37 ^∘^C in PBS. Each condition was centrifuged for 5 min at 1500 rpm at 20 ^∘^C and resuspended in 1 mL of fresh medium. The different concentrations and combinations were optimized so that even after two cell divisions, the barcodes will be different enough to differentiate the cell clones. Cells were plated in a 6-well plate and kept in culture conditions until sorting (in an incubator 37 ^∘^C, 5% CO_2_). Cells were were stored at 37 ^∘^C throughout the sorting process and sorted at 20 PSI through a 100-*µ*m nozzle on an FACS AriaII (BD). The sorting strategy was done using single-labeled cell populations (CFSE, CTY, CTV and negative); then, gating was performed on FSC-A/SSC-A to capture live cells, SSC-H /SSC-A to capture single cells, and CTV positive cells. One cell from each subgroup (6 cells total) was isolated in a well of a 96-well plate which contained 500 non-labeled feeder cells in either self-renewing medium or differentiating medium through a-100 µm nozzle with yield, purity, and phase mask of 0, 32, and 16 respectively (single-cell mask). A well then contained 6 mother cells, each one labeled with a unique fluorescent barcode and the feeder cells. The plate was then put back in culture conditions (in an incubator 37 ^∘^C, 5% CO_2_).

CTFR (Cell Trace Far Red Proliferation kit (Thermo Fisher Scientific)) labeling was performed 20 h after mother cell sorting, in the plate, so that the cells had time to divide once. The staining was made as heterogeneous as possible, thanks to the feeder cells but also by using very low concentrations of dye and for a very short amount of time. Indeed, 0.37 *µ*M of CTFR (Cell Trace Far Red Cell Proliferation kit (Thermo Fisher Scientific)) was added to each sample (in approximately 50 *µ*L of medium), and then 100 *µ*L of medium was added to dilute the dye. The plate was centrifuged for 5 min at 200G, then 120 *µ*L of medium was removed, and 50 *µ*L of new medium added to each labeled well. This heterogeneous CTFR staining will allow to discriminate the next division meaning within the 4 cousin cells and how they are paired two by two. Indeed, each daughter cell will receive a unique intensity of CTFR dye which will be discriminating after one more cell division. Cells were kept in culture conditions for an additional 20 h (in an incubator 37 ^∘^C, 5% CO_2_).

On the third day, after the second division, the content of the wells containing the cousin cells were transferred into polypropylene FACS tubes and briefly vortexed immediately before loading into the sorter. The sorting strategy was done using single-labeled cell populations (CFSE, CTY, CTV, CTFR and negative); then, gating was performed on FSC-A/SSC-A to capture live cells, SSC-H /SSC-A to capture single cells, and CTV positive corresponding to the second division peak and exclude feeder cells. Cells were sorted on a FACS AriaII (BD) at 20 PSI through a 100-*µ*m nozzle with yield, purity, and phase mask of 32, 16, and 0 respectively, in PCR tubes containing lysis buffer (0.2% Triton (Sigma Aldrich), 0.4 U/$\mu$L RNaseOUT (Thermo Fisher Scientific), 400n M RT primers (Sigma Aldrich)), and scRNA-seq primers. The fluorescent intensities for CFSE, CTY, and CTFR were recorded for each cell to further reconstruct relationships between the cells using our clustering algorithm.

#### Cousin cell identification

Clustering was performed using the R mclust package [30] (version 5.4.10-https://gitbio.ens-lyon.fr/LBMC/sbdm/sister-cells commit 76615c6e). This clustering script finds the genealogical relationships between cells in two steps. First, cousin cells are grouped together by their fluorescent barcode, determined by the CFSE and CTY fluorescent intensity values. Thus, if two, three, or four cells have the same CFSE and CTY intensities levels, they will be considered as cousins. In a second step, we select the groups for which the 4 cousin cells were sorted in the plate; then, the program identifies the two pairs of sisters within the 4 cousins. To do this, the median CTFR intensity is calculated; then, the two cells that have intensity values higher than the median are matched, and the other two that have lower intensity values are matched together. Finally, when sorting, we used an index sorting option, which allows us to know in which well of the plate each cell was sorted. With this position information, our analysis program returns the position of the retained cells, i.e., the cells belonging to the cousin groups for which the 4 cells were successfully isolated in the lysis plate.

### scRT-qPCR data generation

#### scRT-qPCR one step

Lysed cells were heated at 65 ^∘^C during 3 min for hybridization with RT primer and immediately transferred into ice. Seven microliters of RT-PCR mix (Superscript III RT/Platinum Taq 0.1 *µ*L (Invitrogen), reverse and forward primers and spikes at 1.33 *µ*M final concentration and homemade 2X reaction Mix (120 mM Tris SO4 pH = 9, 2.4 mM MGSO4, 36 mM (NH_4_)_2_SO_4_, 0.4 mM dNTP)) was added to each well before launching of reverse transcription and PCR run on thermocycler (program : 50 ^∘^C 15 min–95 ^∘^C 2 min–20 cycles 95 ^∘^C 15 s/60 ^∘^C 4 min–hold 4 ^∘^C). Three microliters of exonuclease mix (exonuclease I 1.6 U/mL (NEB), exonuclease buffer 1X (NEB), nuclease-free water (Thermo Fisher Scientific)) was added, and samples were incubated for a digestion run on thermocycler (program : 37 ^∘^C 30 min–80 ^∘^C 10 min). Pre-amplified samples were diluted five times in TE low EDTA (10 mM Tris, 0.1mM EDTA, pH = 8) and kept at − 20 ^∘^C for one night before qPCR.

#### qPCR with Fluidigm Biomark technology

3.15 *µ*L of pre-amplified samples were distributed into a 96-well plate, and 3.85 *µ*L of qPCR mix (Sso EvaGreen Supermix with Low ROX (Bio-Rad)+ 20X DNA binding dye sample loading reagent) was added to each well. Simultaneously, a 96-well plate with primer mix (forward and reverse primers and spike at 2 *µ*M final concentration, 2X Assay Loading Reagent, TE low EDTA) was prepared. The microfluidigm chip was primed with injection oil using the IFC Controller HX (Fluidigm). Five microliters of primers and 5 *µ*L of samples were loaded in the dedicated wells of the chip. Air bubbles were removed with a needle. Samples and primers were mixed in the IFC Controller HX (Fluidigm) with the loading program. The chip was then transferred in the Biomark HD system (Fluidigm) for qPCR with “HE 96x96 PCR+Melt v2.pcl” thermal cycling protocol with auto exposure.

#### Quality control and normalization

Ct values obtained from the Biomark HD System (Fluidigm) were exported as excel files and quality control was manually done. For each gene, “failed” quality control readings identified by the Fluidigm software were removed. Four negative controls (mix of water and lysis buffer) were used to detect unwanted amplification, and the associated genes were also removed. Finally, two externally added controls (spike 1 and spike 4, Fluidigm) were used to control amplification consistency. Filtered data frame was then imported into R (version 4.2.0) for normalization to remove amplification bias (https://gitbio.ens-lyon.fr/LBMC/sbdm/sister-cells commit 45a65972). For each cell, expression values were calculated by subtracting the gene Ct value from the geometric mean of Ct values from spike 1 and spike 4 of the corresponding well. Then, an arbitrary differential cycle threshold value of − 22 for null signal (corresponding to a Ct value of 30) was assigned for all genes with a Ct value less than − 22.

### scRNA-seq data generation

#### scRNA-seq library preparation

Subsequently to sister cell or cousin cell isolation, we performed single-cell RNA sequencing (scRNA-seq) using a modified version of the Mars-seq protocol [31] published in [23]. This specific protocol of scRNA-seq allowed us to know in advance which cell barcode would be carried by each cell and thus preserving the genealogy information of the cells. Briefly, reverse transcription (RT) was performed so every mRNA of the cells were tagged with a combination of unique cell barcode and an 8-bp random UMI (unique molecular identifier) sequence for further demultiplexing. After barcoding, all mRNA were pooled, and second DNA strand was synthetized. Amplification was done overnight using in vitro transcription (IVT) to obtain a more linear amplification. A second barcode was added by RT to identify plates. Libraries were amplified by PCR, and Illumina primers were added.

#### Sequencing

Libraries were sequenced on a Next500 sequencer (Illumina) with a custom paired-end protocol (130 pb on read1 and 20 pb on read2) and a depth of 200,000 raw reads per cell.

#### Data preprocessing

Fastq files were pre-processed through a bio-informatics pipeline developed in our team on the Nextflow platform [32], available in https://gitbio.ens-lyon.fr/LBMC/sbdm/mars_seq and published in [23]. Briefly, the first step removed Illumina adaptors. The second step de-multiplexed the sequences according to their plate barcodes. Then, all sequences containing at least 4T following the cell barcode sequence and UMI sequence were kept. Using UMItools whitelist, the cell barcodes and UMI sequences were extracted from the reads. The cDNA sequences were then mapped on the reference transcriptome (Gallus GallusGRCG6A.95 from Ensembl), and UMIs were counted. Finally, a count matrix was generated for each plate.

#### Quality control and data filtering

All analysis were carried out using the R software (version 4.1.2 [33]) and are available on the following git repository https://gitbio.ens-lyon.fr/LBMC/sbdm/sister-cells. For the sister cell dataset, cells were filtered based on several criteria: reads number, genes number, counts number, and ERCC (External RNA Controls Consortium) content. For each criteria, the cut off values were determined based on SCONE [34] pipeline and were calculated as follows:

Mean(parameter) − 3*sd(parameter)

We then removed orphan cells, meaning cells which sister was not present in the dataset. After filtering, we kept 60 undifferentiated cells (30 couples) and 64 differentiating cells (32 couples). For the cousin cell dataset, we performed the same filtering strategy and kept only cell groups which contained the 4 cousin cells. After filtering, we kept 32 undifferentiated cells (8 groups of cousins) and 20 differentiating cells (5 groups of cousins).

Based on [35] work, we applied a stringent filter on gene expression level. Genes were kept in the dataset if there were expressed on average across all the cells. For instance, in a dataset containing 100 cells, the filtering threshold would be of 100 UMIs for the gene to be kept. After applying this filter, we kept 1177 and 983 genes for the sister cell dataset and the cousin cell dataset respectively.

#### Normalization

Filtered matrix were normalized using SCTransform from Seurat package (version 1.6 [36]-https://gitbio.ens-lyon.fr/LBMC/sbdm/sister-cells commit 945aaca7 and 94f13467) and were corrected for batch effect, day of isolation effect, medium effect, and sequencing depth effect. Both datasets (sister cells and cousin cells) were processed independently.

### Bioinformatics analysis on R

All analysis were carried out using the R software [33] (version 4.1.2 for T2EC and version 4.2.0 for CD34+). Plots were performed ggplot2 package (version 3.3.6).

#### Dimensional reduction

Uniform Manifold Approximation and Projection (UMAP) dimension reduction and visualization were performed using UMAP package (version 0.2.8.0 [37]). Principal component analysis (PCA) was done using R prcomp function with default parameters.

#### Distance computation

Distances were computed on normalized matrix between all cells using the dist function from R software. Distances between sister cells were extracted and compared to the same number of randomly chosen distances of non-related cells. One thousand subsamplings were performed this way (https://gitbio.ens-lyon.fr/LBMC/sbdm/sister-cells commit 8417545d and 45a65972).

#### Statistical tests

Mean comparisons were performed using Student *t*-test or Wilcoxon test when Student *t*-test was not applicable. It was decided after testing the normal distribution of the variables using a Shapiro-Wilk test.

#### Linear model with random variable and mixed effects model

Linear model with random variable and mixed effects model analysis were performed using lme4 R package (version 1.1-29-https://gitbio.ens-lyon.fr/LBMC/sbdm/sister-cells commit c24fa472). The models were defined as followed:

Mixed effect model definition:

$$\begin{aligned} Y = p1 + p2 + e \end{aligned}$$

Linear model with random variable definition :

$$\begin{aligned} Y = p2 + e \end{aligned}$$

where *Y* is the mean expression of each gene, p1 is the fixed effect, and p2 is the random effect. Here, p1 corresponds to the biological condition and can take two values (undifferentiated and differentiating), and p2 is the sorority effect. Two sister cells have the same discrete value. And *e* is the error of the model. Null models are the above model but without the random effect, e.g., the sorority effect. Genes were selected based on a significant adjusted Benjamini-Hochberg (BH) *p*-value after performing a likelihood ratio test between the model and the null model.

## Results

### Cellular models of differentiation

To consolidate our results, we used two different cell differentiation models.

As a first model, we used primary human cord blood derived CD34+ cells. These cells are believed to be a mixture of so-called multipotent progenitors and stem cells that retains the capacity to differentiate into various cell types. Under ex vivo conditions, the CD34+ cells, unless stimulated, are stopped in the cell cycle and survive only a few days. When stimulated with a mixture of cytokines, they re-enter the cell cycle and will differentiate into two different committed progenitors [15]. Briefly, by 24 h after stimulation, a burst in transcription produces a mixed transcription profile called “multilineage primed” state [11], and by the end of the first cell cycle (between 40 and 60 h), cells with two different transcription profiles emerge in the population [15, 38]. However, this first fate decision is a highly dynamic and fluctuating process which is more complex than a simple binary switch between 2 options [15]. In the present work, we investigated by scRT-qPCR the transcriptional profile of couples of CD34+ sister cells derived from the first cell division after the cytokines stimulation.

As a second model, we used chicken primary erythrocytic progenitors called T2EC [28]. Contrary to the human cord blood CD34+ cells, these cells can be maintained in a self-renewing state in vitro under appropriate culture conditions [29]. They can be induced to differentiate at will into mature erythrocytes by a change of medium [29]. The T2EC cells undergo a simple “switch”: they leave the self-renewing phase and enter a differentiation trajectory without bifurcation toward different end point phenotypes. This model allows a direct comparison of related cells in two different states: self-renewing and during differentiation. Furthermore, a previous study on this model had highlighted a critical point of cell commitment, 24 h post-differentiation induction characterized by the rise in gene expression variability, measured with entropy [14]. Thus, we focused on the first steps of T2EC differentiation and generated two independent datasets, the first one to investigate the transcriptional profile of couples of generation 1 sister cells in both cellular states and the second to investigate families of generation 2 sister and cousin cells in both state by a scRNA-seq approach.

### Cell isolation

#### Isolation of first generation cells

**Fig. 2:**
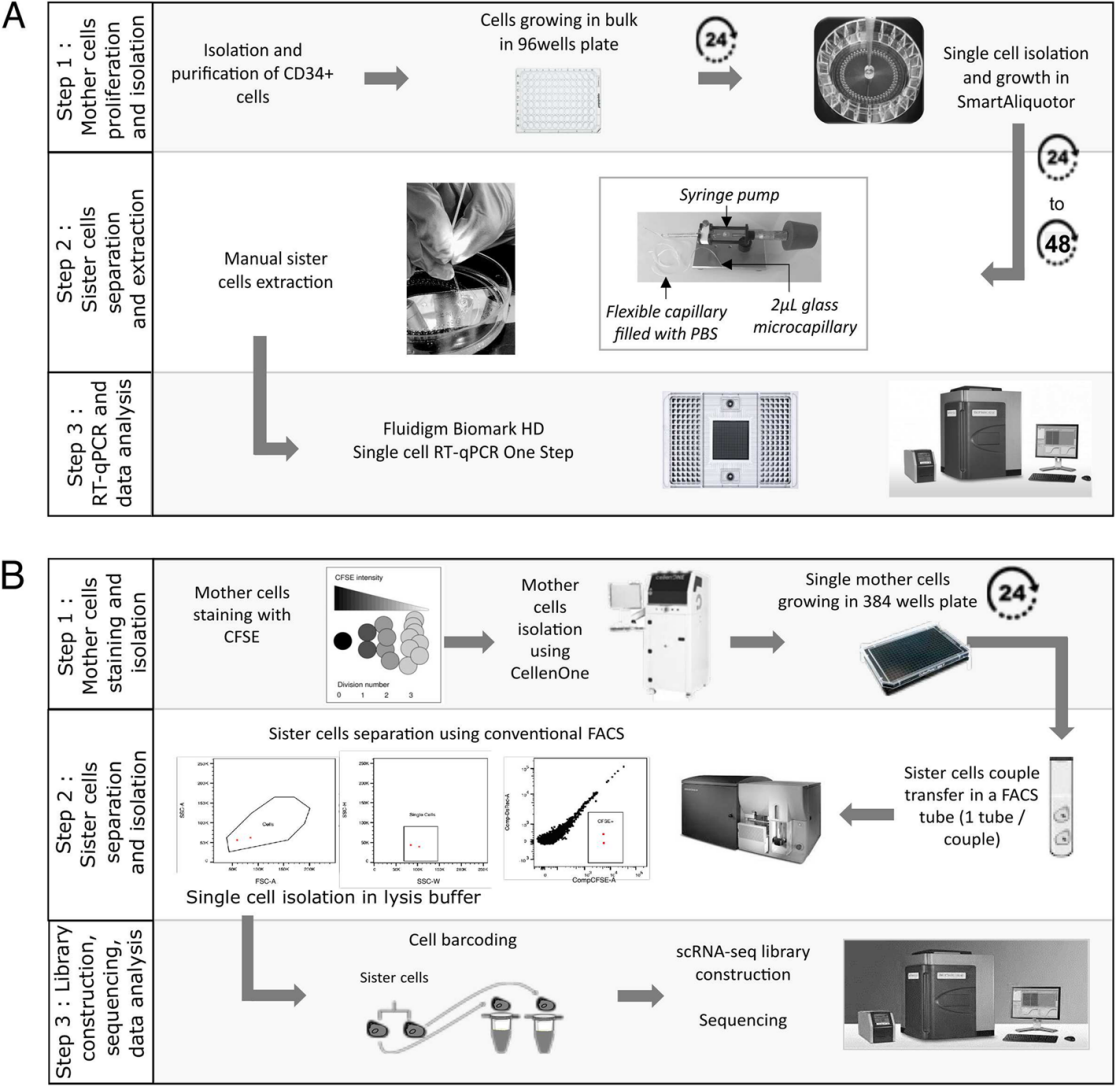
General workflows developed to generate, follow and separate generation 1 sister-cells from CD34+ (A - manual strategy) or T2EC (B - cytometry-based strategy) mother cells. See text and Methods for details

We achieved the technical challenge to isolate related cells following their first and second division (generation 1 sister cells and generation 2 sisters and cousin cells). We first developed two different methods to recover generation 1 sister cells, depending upon the cellular model at hand: a manual one and a cytometry-based method. Those original strategies are presented below and in Fig. 2. The technical details are explained in the “Methods” section.

Human CD34+ cells were grown during 24 h in a standard 96-well plate before being isolated into single cells, using a Smart Aliquotor device in which individual cells still share the same medium. Isolated mother cells were then cultured for 24 to 48 h in the device to allow one cell division. The wells were regularly inspected to detect this first division. Then, the resulting sister cells were isolated manually under a microscope using a pressure controlled microcapillary and recovered in lysis buffer for further processing. The cells transcriptomes were analyzed by single-cell quantitative RT-PCR using the Fluidigm system as described in [15].

Manual hand-picking was not applicable to our other cell model, T2EC, since these cells tend to stick to each other even long after the mitosis is over. We thus decided to isolate T2EC sister cells using FACS to favor a fast isolation. T2EC mother cells were isolated after CFSE staining using CellenOne®low-pressure cell sorter and plated in a 384-well plate. Cell doublets, resulting from the first division, were identified using an inverted microscope. The two cells were then isolated using a FACS Aria cytometer and recovered directly in tubes containing lysis buffer and scRNA-seq primers, for which the cell barcodes sequences were known in advance. scRNA-seq libraries were then constructed as previously described in [23] and sequenced.

Successfully recovering the two sister cells using FACS is *per se* a remarkable achievement, as this method usually requires hundreds of cells to start with, whereas the initial population here consisted of two cells. To achieve this, we first used the CFSE fluorescence intensity to ensure that the objects isolated were indeed cells (Additional file 1: Fig. S1A–B for self-renewing medium and C–D for differentiating medium). CFSE stably binds to the amine groups present in cytoplasmic proteins, conferring stable fluorescence intensity to the cell. As total protein content is supposed to be relatively equally distributed between sister cells during cell division, so is the fluorescence intensity [39, 40]. We used this specification to validate that the two cells isolated were actually sister cells. We evaluated the CFSE intensity correlation between pairs of sister cells and compared it to intensity correlation values of randomly paired cells from the same dataset (Additional file 1: Fig. S1E–F for self-renewing cells and G–H for differentiating cells). Outstandingly, CFSE correlation values between self-renewing sister cells and differentiating sister cells were extremely high (0.91 and 0.95 Additional file 1: Fig. S1E and G, respectively), whereas for randomly paired cells, CFSE correlation values dropped between − 0.07 for self-renewing cells and 0.18 for differentiating cells (Additional file 1: Fig. S1F and H, respectively) indicating no correlation. Those results validated that our general strategy did allow to retrieve accurately generation 1 sister cells. The same procedure was applied to generation 1 T2EC mother cells in proliferating phase and in differentiation by sorting the mother cells either in self-renewing medium or in differentiation-promoting medium.

We further analyzed the T2EC scRNA-seq data quality and reproducibility by characterizing the observed biological process applying two dimensional reduction methods, UMAP and PCA (see the “Methods” section). As expected, the cells separated based on their differentiation state (Additional file 1: Fig. S2A and B, respectively). This observation was validated by a differential expression analysis between the two groups (self-renewing and differentiating cells-Additional file 1: Fig. S2C). Genes involved in early erythrocyte maturation and inhibition of differentiation such as *ID2* known to be an erythropoiesis inhibitor in mice [41] and *FTH1* and *TMSB4X* known to be expressed in human erythroid progenitors [42] were upregulated in self-renewing cells, while *HBBA*, *HBAD*, and *HBA1* genes involved in hemoglobin complex and *TAL1*, erythroid differentiation factor, were upregulated in differentiating cells, as previously described [23].

#### Isolation of second generation cells

**Fig. 3:**
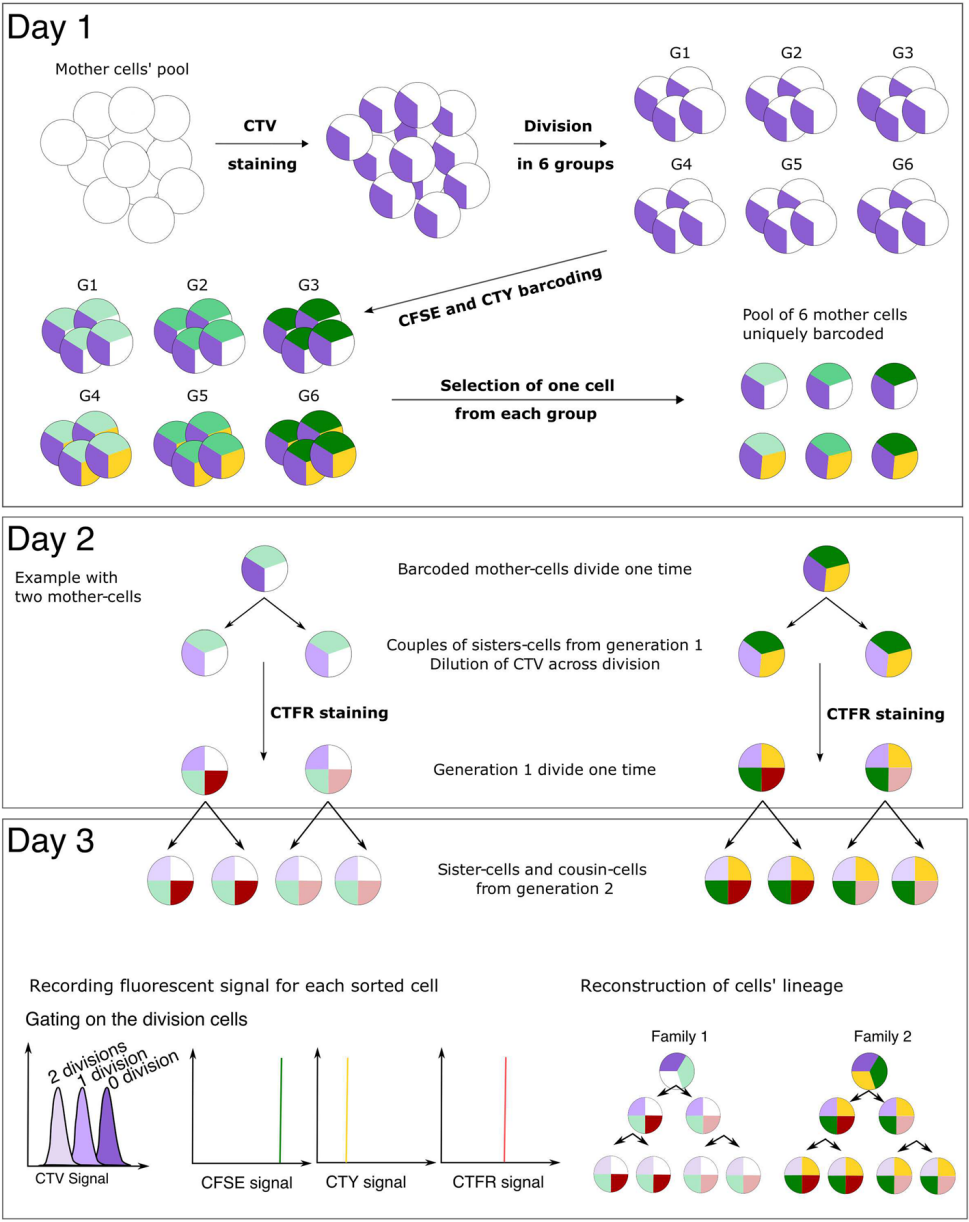
General labelling strategy for generation 2 T2EC cells identification. On day 1, a population of mother-cells was stained using CTV. The CTV positive population was divided into six subgroups, and each subgroup was uniquely barcoded using a combination of CFSE and CTY concentrations, resulting in six distinct fluorescent barcodes. One mother-cell from each subgroup was then retrieved and combined in a well for approximately 24 hours of culture (resulting in a total of six mother cells, each with a unique fluorescent barcode). On day 2, following the first division, a fourth dye, CTFR, was introduced to label sister-cells with a different intensity in order to be able to discriminate the cells relationship after the subsequent division. On day 3, cells which underwent 2 divisions, determined by the intensity of CTV, were sorted into single-cells, and their fluorescent intensities for CTY, CFSE and CTFR signals were recorded. Finally, a dedicated script was used to infer the relationships of cells based on the fluorescent intensities (see "Methods" section)

Using the T2EC model, we then developed another FACS sorting methodology to generate a second dataset consisting of generation 2 sister and cousin cells, that is to say the 4 cells resulting from two divisions, both in self-renewing state or in differentiation state. To record cells genealogies, we used different cell tracers to achieve fluorescent barcoding of cells families, and we stained the cells sequentially to retrieve both cousin relationships and sister relationships within different families (Fig. 3). Briefly, a small number of mother cells was stained such as every mother cell carried a unique fluorescent barcode. Each fluorescent barcode consist in a combination of CTY and CFSE at different intensities, leading to 6 different barcodes. This barcode is passed along to the mother cell progeny over two cell generations to allow a good discrimination of cells families. One mother cell from each barcode was isolated by FACS in a single well of a culture plate. After the first cell division, another cell tracer was added to discriminate sister cells within the cousin groups. After the second cell division, the cells (generation 2) were sorted in lysis buffer containing scRNA-seq primers of known sequence, and the relationships between the cells were recovered using a clustering script developed in our team. Details of the methodology are presented in Fig. 3 and in the “Methods” section. Further viability analysis was performed and showed that the staining strategy did not compromise cells physiology (Additional file 1: Fig. S3).

Using first generation methodologies, we successfully collected 86 CD34+ cells, 60 self-renewing T2EC cells, and 64 differentiating T2EC cells encompassing respectively 43, 30, and 32 couples of generation 1 sister cells. With the second generation original fluorescent barcoding approach, we collected 8 families of generation 2 self-renewing T2EC cells (32 cells) and 5 families of generation 2 differentiating T2EC cells (20 cells).

### Strategy to evaluate transcriptomic similarities between related cells

We used the Manhattan distance as the principal metric to assess global transcriptomic similarities between cells. Manhattan distance is a robust geometric distance that exhibits low sensitivity to data sparsity, which is inherent to single-cell transcriptomics data [43]. Euclidean distance was also used to confirm the reliability of the results.

In the case of hypothesis 1, which concerns memory maintenance, there would be no significant transcriptional differences between self-renewing sister cells compared to differentiating sister cells. This hypothesis would imply that at the first cell generation, differentiating sister cells would exhibit a similar distance from each other compared to self-renewing sister cells. And at the second generation, there would be no difference either between differentiating sister cells compared to self-renewing sister cells nor between differentiating cousin cells compared to self-renewing cousin cells.

In the case of hypothesis 2, which pertains to the gradual erasure of memory, there would be a gradual increase in the sister-to-sister differences as differentiation proceeds. Meaning, at the first generation, differentiating sister cells would present a greater distance compared to self-renewing sister cells. As the second generation is reached, this distance would increase and would be supported by (1) second generation differentiating sister cells presenting a greater distance compared to second generation self-renewing sister cells and (2) second generation differentiating cousin cells presenting a greater distance compared to self-renewing cousin cells.

In the case of hypothesis 3, which suggests an instantaneous erasure of memory, there would be very strong transcriptional differences between self-renewing and differentiating sister cells at the beginning of the differentiation process, with no evolution of those differences thereafter, that is, at the first generation, differentiating sister cells would present a substantial greater distance between them compared to self-renewing sister cells. Upon reaching the second generation, differentiating sister cells would display a similar or smaller distance compared to self-renewing sister cells, and differentiating cousin cells would present a similar or slightly greater distance compared to self-renewing cousin cells.

### Transcriptomic similarities between generation 1 sister cells after one division

We started by assessing whether or not generation 1 sister cells displayed more similar global gene expression levels compared to non-related cells. Here, non-related cells correspond to cells which do not originate from a common mother cell. The Manhattan distances were computed between the gene expression vectors of each cell. Gene expression vectors for the 43 couples of CD34+ sister cells were composed of 83 genes after quality control and data filtering (see the “Methods” section). Those genes were either selected for their known function in the early differentiation of hematopoietic cells (64% of them) or randomly chosen (36%) to provide an assessment of the overall transcriptional state of the genome [15]. For the 62 couples of T2EC sister cell gene expression vectors, we retained 1177 genes after data filtering and normalization of scRNA-seq data (see the “Methods” section). Feature selection was performed based upon the Breda et al. work [35], showing that in scRNA-seq dataset, genes with too low counts could not be analyzed due to poor variance estimation. We therefore kept genes if the ratio of the number of UMIs for this gene to the total number of cells is at least 1, which means that this gene may not be expressed in some cells and may be more highly expressed in others. The resulting 1177 genes represent 10% of the genes in our dataset.

**Fig. 4:**
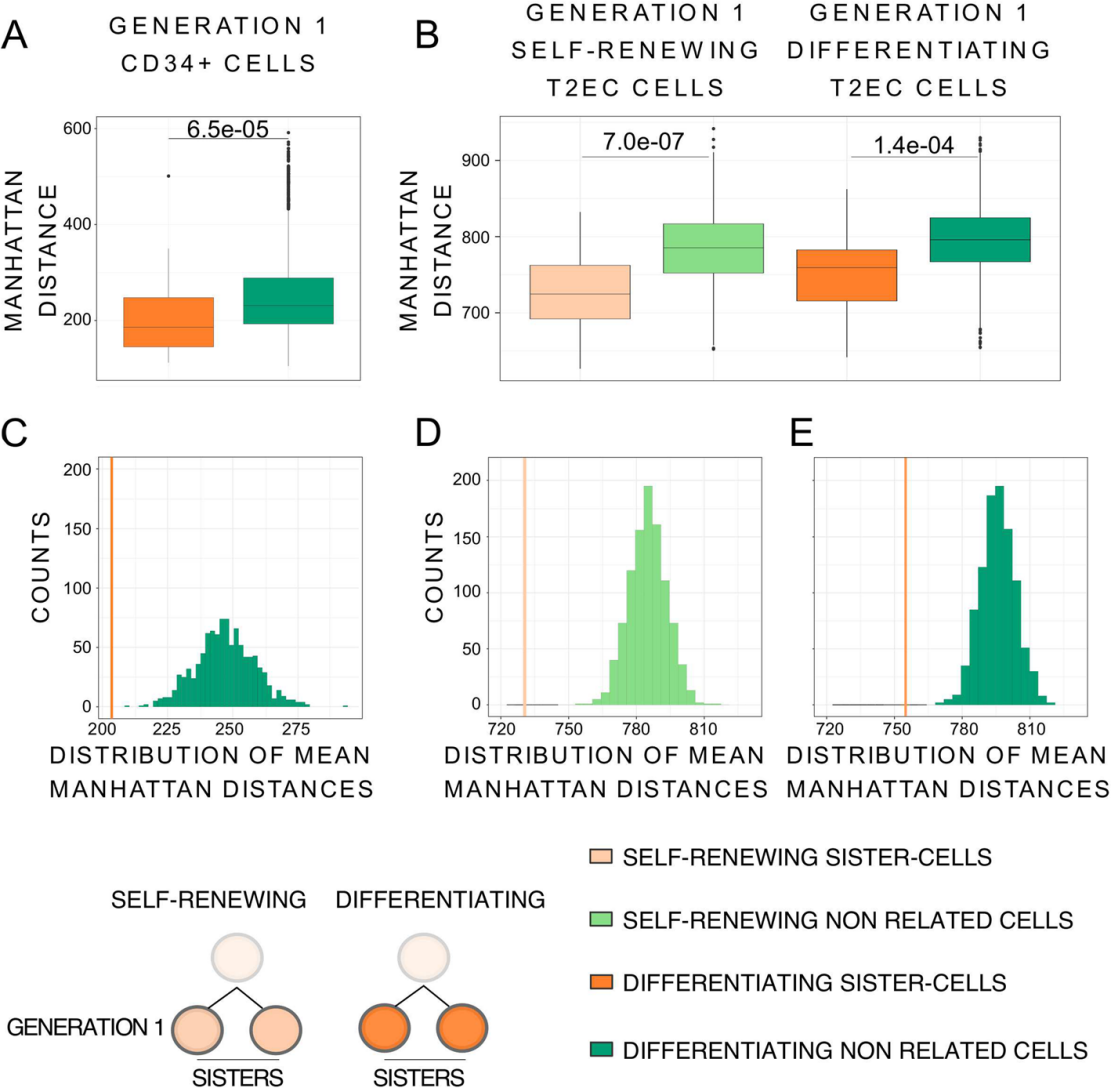
Manhattan distances comparison between generation 1 sister-cells and non related cells. (**A**) Boxplots of Manhattan distances between the generation 1 CD34+ sister and non related cells. CD34+ sister-cells (43 couples) are in orange and CD34+ non related cells (3612 couples) in green. Manhattan distances were computed using all the 83 selected genes. Statistical comparison was performed using Wilcoxon test. (**B**) Boxplots of Manhattan distances between generation 1 T2EC sister and non related cells. Manhattan distances were computed between all cells from the same biological conditions using all the 1177 selected genes. Self-renewing sister-cells (30 couples) are in light orange and self-renewing non related cells (1740 couples) in light green, differentiating sister-cells (32 couples) are in orange and differentiating non related cells (1984 couples) in green. Statistical comparison was performed using Student *t*-test. (**C**) Histograms of mean Manhattan distances of 1000 random subsampling of distances between 43 CD34+ non related cell pairs (green), compared to the mean distance between the 43 CD34+ generation 1 sister-cells pairs (orange line). (**D**) Histograms of mean Manhattan distances of 1000 random subsampling of distances between 30 T2EC self-renewing non related cell pairs (light green histogram), compare to the mean distance between the 30 T2EC self-renewing generation 1 sister-cells pairs (light orange line). (**E**) Histograms of mean Manhattan distances of 1000 random subsampling of distances between 32 T2EC differentiating non related cell pairs (Green histogram), compare to the mean distance between the 32 T2EC differentiating generation 1 sister-cells pairs (orange line)

We performed the analysis by computing the Manhattan distances between generation 1 sisters and randomly selected non-related cell pairs from the same pool of cells (Fig. 4A, B). Mean distances were then compared between the two groups (generation 1 sisters and non-related cells) for both CD34+ and T2EC cells. For the latter, both self-renewing and differentiating cells were analyzed separately. For both models and in both biological conditions, mean Manhattan distances between generation 1 sister cells were always significantly smaller than the mean distances between non-related cells (Fig. 4A, B-Wilcoxon test for CD34+ cells *p*-value = 6.5e−05, Student *t*-test for self-renewing T2EC cells *p*-value = 7e−07 and for differentiating T2EC cells *p*-value = 1.4e−04). Similar results were obtained using Euclidean distances (Additional file 1: Fig. S4A and B).

To ensure that the difference in mean distance observed between generation 1 sisters and non-related cells was not an artifact due to difference in sample size, we performed a randomization experiment with multiple subsamplings. Briefly, 43 non-related CD34+ cell pairs, 30 non-related self-renewing T2EC cell pairs, and 32 non-related differentiating T2EC cell pairs were randomly drawn from the corresponding groups 1000 times. The mean distance was calculated for each pair and plotted on the histograms shown on Fig. 4C, D, E. For both models, and for T2EC in both biological conditions, the mean distance between generation 1 sister cells was never part of the non-related cells mean distances distribution. Similar results were obtained using Euclidean distances (Additional file 1: Fig. S4C, D and E). Those results strongly suggest that the observed difference was genuine and not due to sampling bias.

This is a clear indication that the gene transcription profiles of generation 1 sister cells in both experimental models are more similar to each other than to those of non-related cells of the same type sharing the same environment and undergoing similar biological processes. Those results also highlight that differentiating sister cells from generation 1 display a form of transcriptional memory, which complements previous studies demonstrating a transcriptional memory in self-renewing sister cells.

Focusing on the T2EC model, for which we compared related cells in two cellular states (self-renewing and differentiating), although the difference was borderline non statistically significant (*p*-value = 6e−02), our results point toward a decrease in transcriptome similarity during differentiation as shown by a higher mean distance value for generation 1 differentiating T2EC sister cells compared to self-renewing T2EC sister cells.

We wondered whether or not the sister-to-sister cell distance will continue to increase as the differentiation proceeds in the T2EC cells, one generation later.

### Generation 2 cells transcriptomes continue to diverge during differentiation

We generated a second dataset consisting of generation 2 T2EC sister and cousin cells (after two cell divisions) using the methodology described above. As scRNA-seq requires the lysis of the cell under investigation, generation 1 data and generation 2 data consist of different cell families and thus cannot be compared to each other so both dataset were treated and analyzed separately (see the “Methods” section).

The second generation dataset was composed of 4 cousin cells per family (8 families of cells in self-renewing and 5 families of cells in differentiation condition), and within the 4 cousins, they consisted of two couples of sister cells. After data filtering and normalization, we retained 983 genes for subsequent analysis. As a reminder, Manhattan distance is an additive formula; the absolute distance value is thus directly dependant of the number of genes used in its calculation. It is therefore not surprising that the Manhattan distance values are different from generation 1 dataset and generation 2 dataset.

**Fig. 5:**
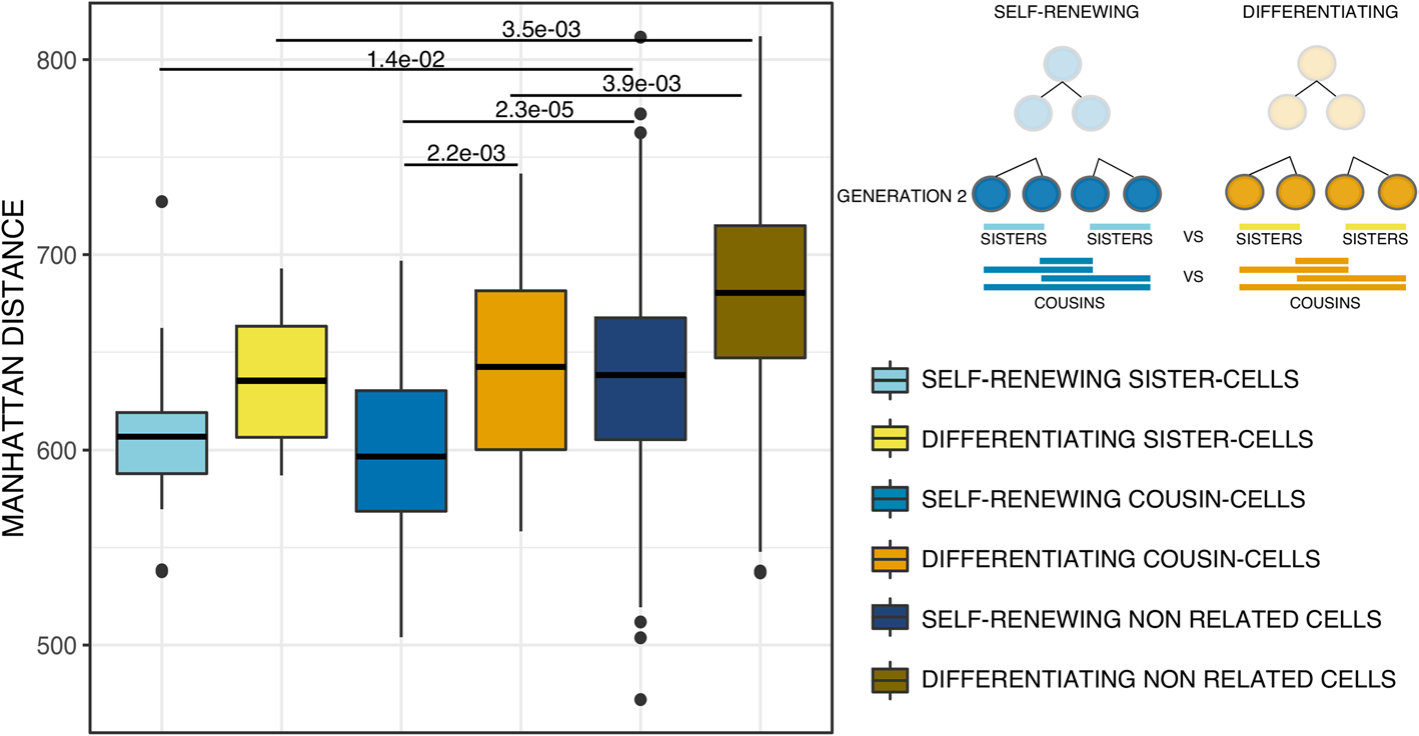
Manhattan distances comparison between generation 2 sisters, cousins and non related T2EC cells. Boxplots of Manhattan distances between generation 2 sisters, cousins and non related T2EC cells. Manhattan distances were computed between all cells (32 self-renewing and 20 differentiating cells) from the same biological condition using the 983 selected genes. Self-renewing generation 2 sister-cells (16 pairs) are presented in light blue, self-renewing generation 2 cousin-cells (32 pairs) are in medium blue and self-renewing non related cells (448 pairs) are in dark blue. Differentiating generation 2 sister-cells (10 pairs) are in yellow, differentiating generation 2 cousin-cells (20 pairs) are in orange and differentiating non related cells (160 pairs) are in brown. Statistical comparisons were performed using Student *t*-test

The analysis of mean Manhattan distances from generation 2 dataset showed that, when comparing conditions, in line with previous results described after one cell generation in Fig. 4, generation 2 differentiating sister cells were less close to each other than generation 2 self-renewing sister cells, although not significantly so (Fig. 5).

Although the differentiating generation 2 sister and cousin cells have a similar average Manhattan distance, this is not inconsistent with our hypothesis because all these cells were induced to differentiate for 48 h. They represent the same generation and not a complete family tree. Indeed, the generation 2 sister cells cannot be used as a proxy of generation 1 sister cells (since they have been differentiating for 48h, and not 24h as the kinetic should be), and thus, however tempting, comparing generation 2 sister cells to generation 2 cousin cells will not give a temporal information as one might intuitively think. For this reason, we did not compare generation 2 sister cells to generation 2 cousin cells as this comparison do not reveal temporal information of the global process (division and differentiation).

Interestingly, generation 2 differentiating cousin cells were statistically further apart from the generation 2 self-renewing cousin cells. Indeed, the average Manhattan distance between generation 2 differentiating cousin cells was statistically greater than that of generation 2 self-renewing cousin cells further confirming a decrease in transcriptome similarity during the differentiation process (Student *t*-test *p*-value = 2.2e−03).

Finally, generation 2 sister cells, regardless of their biological condition (self-renewing or differentiating for 48 h), were always closer to each other than randomly paired cells (Fig. 5-Student *t*-test for self-renewing T2EC cells *p*-value = 1.4e−02 and for differentiating T2EC cells *p*-value = 3.5e−03). Furthermore, the mean Manhattan distances of the generation 2 cousin cells were also statistically smaller than those of non-related cells for both biological conditions, indicating a proximity of transcriptomes which persisted after one more cell generation in both conditions, observed separately (Student *t*-test for self-renewing T2EC cells *p*-value = 2.3e−05 and for differentiating T2EC cells *p*-value = 3.9e−03.

The same analysis using mean Euclidean distances gave very similar results (Additional file 1: Fig. S5).

### Identification of genes subjected to transcriptional memory

**Fig. 6:**
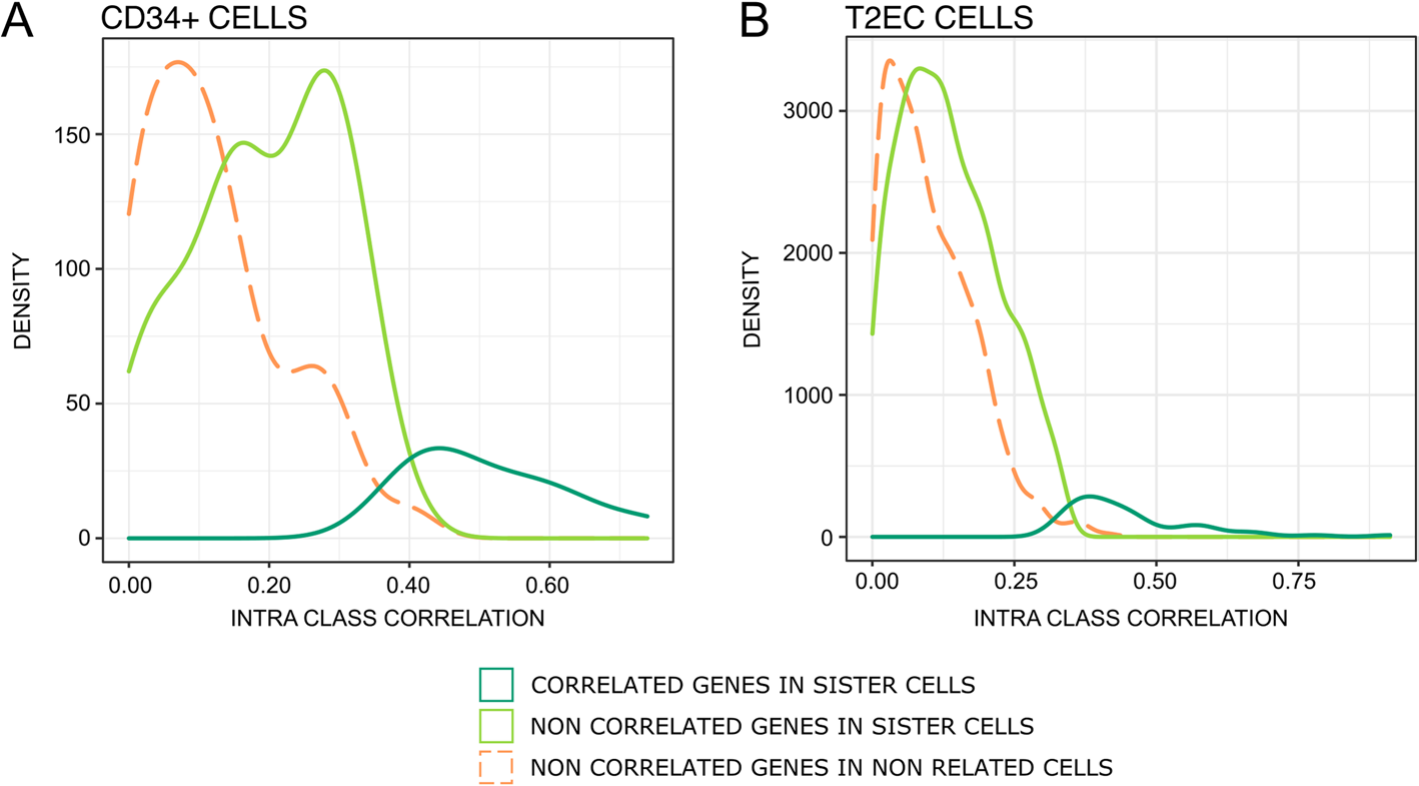
Density plot of genes intra-class correlation in generation 1 sister-cells and randomly paired CD34+ cells (**A**) and T2EC cells (**B**). Identification of memory genes using a linear model with random effect (CD34+) and mixed effect model (T2EC). Memory genes are in dark green (11 genes for the 86 CD34+ cells, 55 genes for the 104 T2EC cells), and non significant genes are in light green (72 for CD34+ cells, 1022 for T2EC cells); no memory genes were identified when cells were randomly paired (orange curve)

We expected that the transcriptomic similarities observed may concern a subset of genes, the “memory genes,” the expression of which would be variable across couples of cells but correlated within couples of sister cells (Fig. 1D). Thus, we applied a “gene-wise” approach to identify genes subjected to transcriptional memory using a linear model with random effect and a mixed effects model. For CD34+ cells, memory genes were identified including a sisterhood random effect to capture between-sisters correlation. For T2EC cells, the expression of each gene was modeled by an additive model combining a fixed condition effect (differentiating or not) to account for difference in expression levels and a sisterhood random effect capturing sister cells correlation. Memory genes were selected by testing for the random effect with a likelihood ratio test comparing the model with and without the sisterhood effect. The test was performed on each gene followed by a Benjamini-Hochberg *p*-value adjustment for multiple testing [44]. As a negative control, we performed the same test on randomly paired cells, and detected no memory gene (Fig. 6).

We detected 10 genes with significant correlation between-sisters in CD34+ cells and 55 genes in T2EC cells (cf. Additional file 1: Table S7). In CD34+ cells, memory genes were involved in diverse functions, including stemness (*GATA1*, *CD38*, *CD133*), differentiation and proliferation (*CD74*, *ERG*, *KIT*), metabolism (*BCAT1*, *HK1*), cytoskeleton (*ACTB*), and tRNA splicing (*C22orf28*). In T2EC, memory genes were involved in erythropoietic differentiation (*HBBA*, *HBA1*, *HBAD*, which are hemoglobin subunits, or *RHAG* membrane channel component involved in carbon dioxide transport), chromosome structure (*SMC2*, *H2AFZ*), ribosomes and translation (*RPS13*, *RPL22L1*, *UBA52*, *EEF1A1*), and metabolism (*GAPDH*, *LDHA*). One should note that *LDHA*, key actors of the glycolytic metabolism, was previously found to also be involved in the erythroid differentiation process [45].

It is quite remarkable that, although many transcription factors were present in the initial list of genes, we found either no (T2EC) or only one (CD34+) transcription factor among memory genes.

We assessed the sensitivity of the Manhattan distance to the gene set size (Additional file 1: Fig. S6). We computed the mean Manhattan distances using random gene subsampling from both datasets (scRT-qPCR and scRNA-seq) and compared the distances between sister and non-related cells. The subsamples are random draws of a percentage of genes from the initial dataset, ranging from 10 to 90%. For each percentage, we performed 1000 random draws of genes and calculated the average Manhattan distance between sisters and non-related cells based on those restricted lists of genes. Subsequently, a Wilcoxon statistical test was carried out. For each percentage, a distribution of the 1000 adjusted *p*-values was plotted (Additional file 1: Fig. S6). The results of the comparisons obtained showed that there is an effect of the sample size: the larger the sample size, the more obvious the sister cells similarity (i.e., the more robust this similarity is to subsampling).

**Fig. 7:**
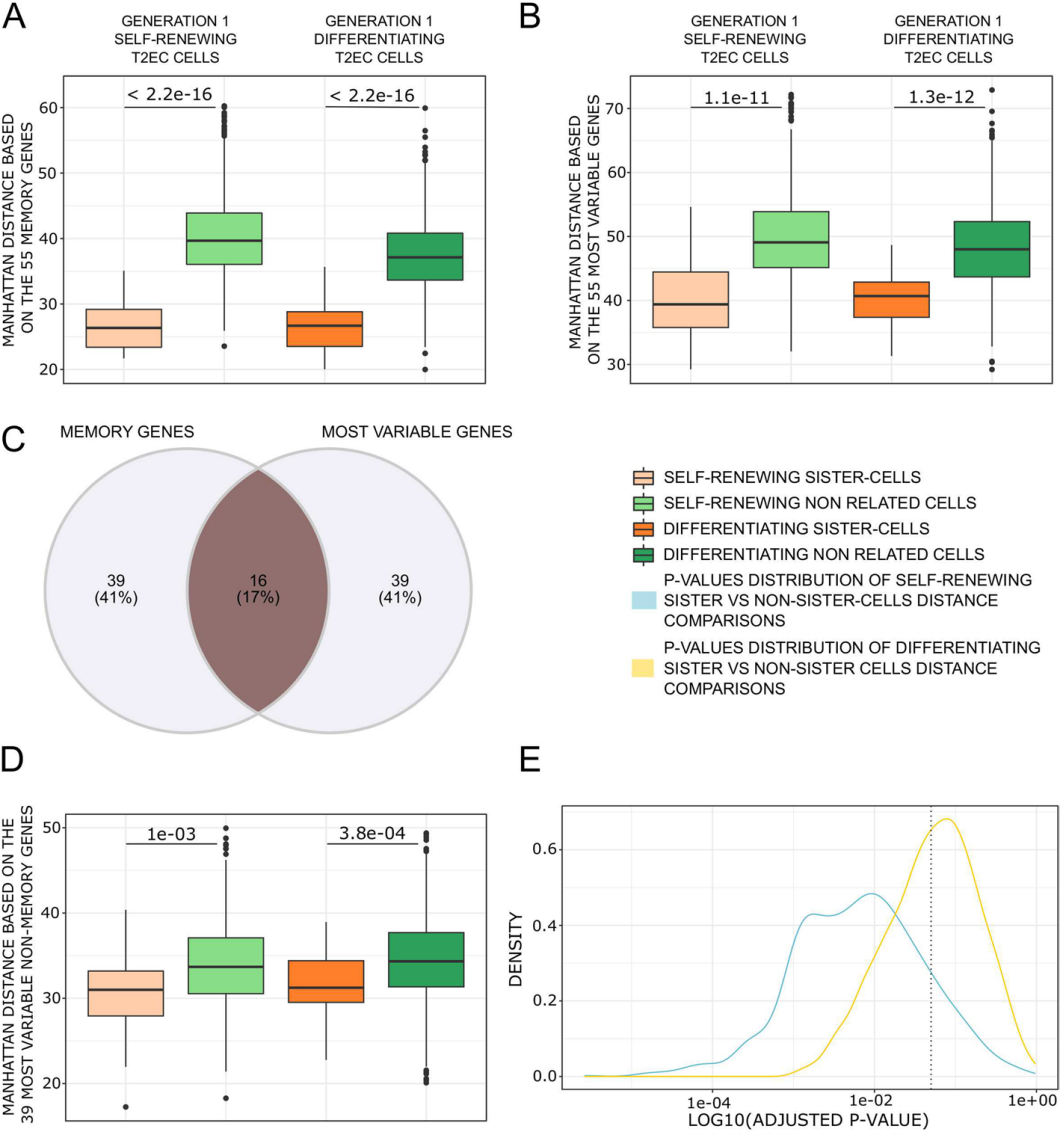
Manhattan distances comparison between generation 1 sisters and non related T2EC cells using subsets of genes. (**A**) Boxplots of the Manhattan distances computed between all cells from the same biological conditions using all the 55 memory genes. (**B**) Boxplots of the Manhattan distances computed between all cells from the same biological conditions using the 55 most variable genes. (**C**) Venn diagram of the 55 memory genes and the 55 most variable genes, 16 genes are common between both categories. (**D**) Boxplots of the Manhattan distances computed between all cells from the same biological conditions using the most variable genes, excluding the memory genes. (**E**) Density plot of the distribution of adjusted *p*-values from Wilcoxon test for mean Manhattan distance comparisons between conditions using 55 randomly draw genes, 1000 times. Blue curve is the *p*-values distribution of mean distance comparison between self-renewing sister-cells vs self-renewing non related cells. Yellow curve is the *p*-values distribution of mean distance comparison between differentiating sister-cells vs differentiating non related cells. *P*-value at 5% is presented as dotted grey vertical line

We then computed again and compared the Manhattan distances for the T2EC cells between sisters and non-related cells using as a vector only the 55 memory genes (Fig. 7A). As a result, the difference in within-distance between sister cells and non-related cells, in both biological conditions (self-renewing and differentiating), was even more pronounced than when computing the Manhattan distances using all 1177 genes of the scRNA-seq dataset (see Fig. 4B). We selected the 55 most variable genes from our dataset and compared the Manhattan distances of sister cells to non-related cells (Fig. 7B). Among the 55 most variable genes, 16 were also memory genes (Fig. 7C). We removed those 16 memory genes from the 55 most variable genes and performed the distance comparison with the 39 most variable genes (Fig. 7D). In both comparisons, a significant difference in within-distance between sister cells and non-related cells in both biological conditions was observed. Finally, we draw 1000 times 55 random genes and compared the Manhattan distances of sister cells to non-related cells. We analyzed the distribution of the corrected *p*-values obtained from the 1000 tests (Fig. 7E). The results showed that there is a probability to observe no significant within-distance difference between sister cells and non-related cells depending on the gene set. This probability is even higher for differentiating cells. But overall, the most significant difference observed was obtained with the 55 memory genes (*p*-value = 2.2e−16), further confirming that the identified genes are the ones imprinted by the transcriptional memory.

The findings revealed that there is a likelihood of encountering no significant within-distance disparity between sister cells and unrelated cells. This likelihood is further elevated for differentiating cells.

**Fig. 8:**
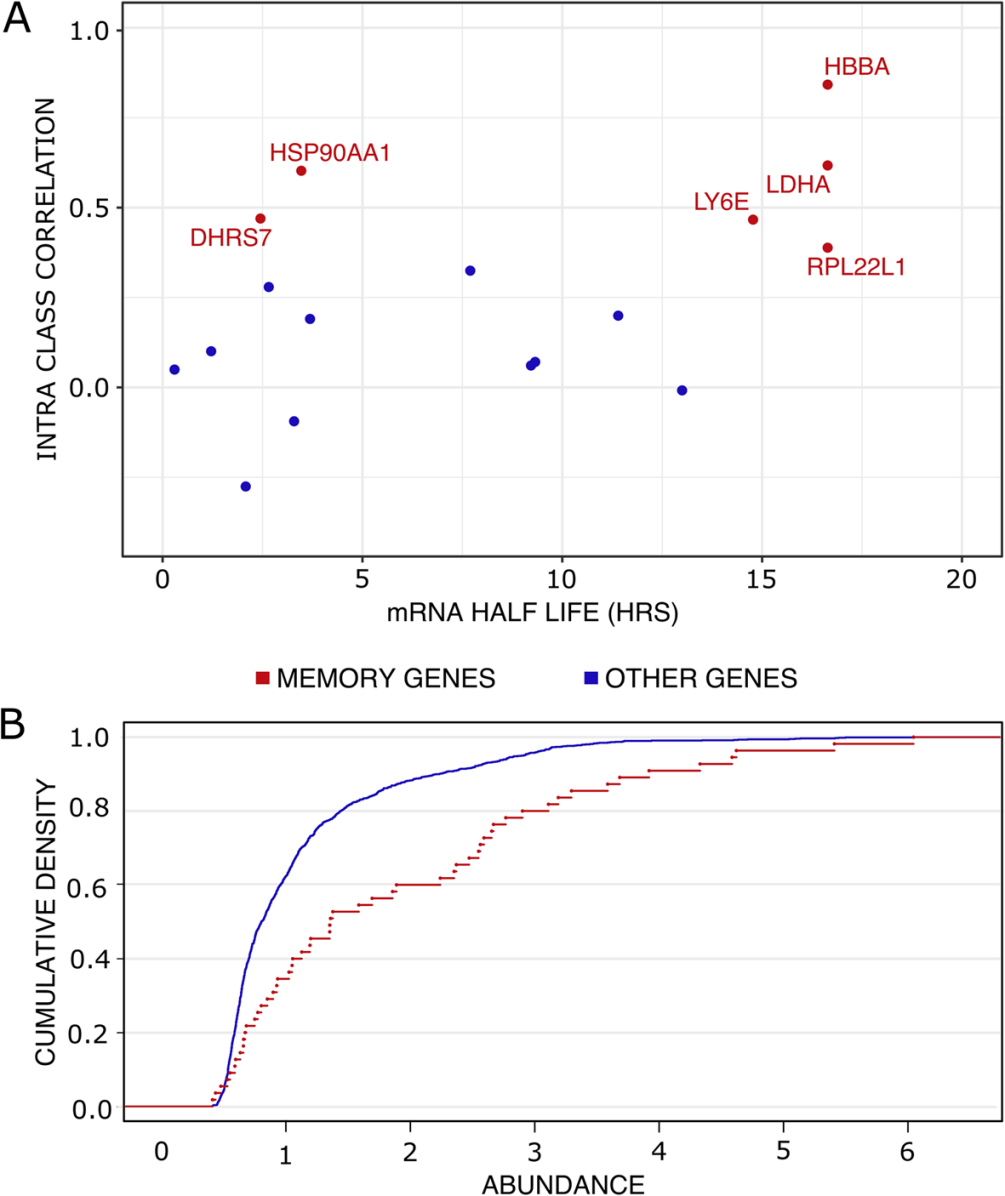
T2EC Memory genes characteristics. (**A**) mRNA half-life of memory genes and other genes present in the scRNA-seq dataset evaluated at 24hrs post differentiation induction [46] vs their Intra Class Correlation value extracted from the mixed effects model. (**B**) Cumulative empirical distribution graph of transcripts abundance of the 55 memory genes in the dataset compared to the total genes (1177) of scRNA-seq data

To validate our findings, we also checked if these memory genes were not only genes associated with high mRNA half-life. We crossed our gene list to a previously published dataset which evaluated half-life duration of genes during T2EC differentiation using scRT-qPCR [46]. We were able to compare the half-life duration of 6 memory genes and found that 4 of them have a relatively long half-life but 2 of them have a quite short half-life (Fig. 8A). Furthermore, other genes with longer half-life were not identified by the model as memory genes. Thus, half-life duration could not be the only cause of memory.

We also questioned the relationship between the level of expression of a gene and its belonging to the memory genes class. One thousand subsample distribution analysis of the abundance of the 55 memory genes compared to the abundance of 55 randomly drawn genes showed an enrichment for higher abundance of the 55 memory genes (Fig. 8B-Kolmogorov-Smirnov test *p*-value = 1.6e−02). We therefore can not exclude that part of the memory is due to high level expression for at least some memory genes and could be related to synthesis and degradation dynamics. However, this result was expected because to prevent false correlation that would be due to high numbers of zeros in expression value of lowly expressed genes between sister cells, we selected genes with mid to high-level of expression in our scRNA-seq data set (see the “Methods” section). Finally, we did not regress cell-cycle effects on our data, due to the fact that cell-cycle is not as well described in chicken cells as it is in mammalian cells and thus cannot exclude that the sister-to-sister resemblance may, in part, be a consequence of the sister cells being at similar state in the cell-cycle. However, while we found a GO term “cell-cycle” enrichment in the 1177 selected genes, no cell-cycle related genes were identified as memory genes, leading us to believe that cell-cycle is not the main driver of this transcriptional memory.

## Discussion

In the present study, we questioned the interplay between the transcriptional memory and the gene expression variability which characterizes differentiation processes.

We developed two experimental frameworks to recover sister cells (generation 1) and one experimental framework to recover cousin cells (generation 2) transcriptomes while preserving the information about their lineage at the resolution of the cell division. We analyzed the transcriptomes of related cells from two different cell differentiation systems using two different single-cell transcriptomics technologies.

Comparison of global transcriptomic state, using Manhattan and Euclidean distances, showed that differentiating generation 1 sister cells (both CD34+ cells and T2EC cells) transcriptomes are globally significantly more similar between each other than between non-related cells.

In our controlled differentiation model (T2EC cells), we observed after one cell division (generation 1), a greater mean distance for differentiating sister cells compared to self-renewing sister cells. Moreover, the difference becomes significant after a second division (generation 2), showed by differentiating cousin cells presenting a significantly higher distance than self-renewing cousin cells. Those results showed that during cell differentiation, related cells deviate faster from each other than during self-renewing divisions.

Mixed models further highlighted that some genes have their expression statistically correlated between sister cells, while none were found between non-related cells. We termed those genes “memory genes” as our results suggest that their expression is more correlated in related cells thus driving the transcriptomic similarity observed between sisters and cousin cells. However, the mechanisms leading to a more correlated expression between related cells for those genes remain to be investigated.

In the introduction, we formulated 3 hypothesis on the possible evolution of the transcriptional memory upon differentiation induction (Fig. 1B). Our results therefore support the second hypothesis: upon differentiation induction, transcriptional memory is gradually erased eventually reconstituting, at the clonal scale, the variability observed in the initial population.

While our experimental methods allow to preserve genealogical cell information for two generations, everything happening later is presently out of reach. We therefore are currently developing a microfluidics-based approach, consisting in a microfluidics chip coupled to scRNA-seq, which could be used on non-adherent cells to investigate cellular memory for several (more than 2) generations [47]. Recently, a study based on a complex cell-tracking system combining time-lapse microscopy, antibody-based cell isolation, and scRNA-seq on robotically-isolated cells has been used to address the question of asymmetric division [26]. While the question is different from ours, the approach could be considered to investigate longer genealogies, but it would require complex equipment and antibodies against chicken cells in order to track division.

In order to explain the existence of memory genes as we (this work) and others [5–8] have described, one need to assume that a significant fraction of those mechanisms must “survive” the mitosis, i.e., be transmitted through the dramatic epigenomic and cellular rearrangements involved in the cell division process. If one assumes that the gene regulatory network (GRN) state is essentially characterized by protein quantities, then it is easy to see that it will be pass through, at least for the proteins with a sufficiently long half life [15]. Reestablishment of the epigenetic marks [48] and of genomic structure [49] after a division process has also been documented.

It has recently been described that the persistence of a low level of transcription throughout the mitosis might at least partly explain how transcriptional memory can be maintained. It would be interesting in that regard, to assess the overlap between our memory genes and these genes for which the mitotic transcription can be detected using UEseq in mitotic chromosomes [50].

Differentiating division is a specific challenge since at each division a subtle combination of changes and stability must be imposed. In this respect, one can see the bookmarking process [51] as a stabilizing process, whereas the increase in gene expression variability [11–19] will affect the GRN state and therefore will tend to modify gene expression burst parameters. In fact, at the single-cell level, gene expression is in essence a probabilistic process that is characterized by a given burst frequency and burst size [52]. The mechanisms regulating this bursting process are still a matter of debate [53, 54], but are usually thought to involve (1) the state of the underlying GRN [55]; (2) the state of the chromatin, a.k.a. the epigenetic marks [7, 8]; and (3) the genomic 3D state [56]. Of course, none of these mechanisms operate in isolation, and more integrated mechanisms, like the metabolism, are also key players in the burst properties of transcription (see, e.g., [57]).

It is interesting to note that our two model systems do behave quite differently in regard to the division process. The initial stages of T2EC erythrocytic differentiation have been shown to result in an increase of the proliferation rate due to a shortening of the G1 period [28]. This is in sharp contrast with the observation that the CD34+ first division occurs after an unusually long cell cycle that lasts on average more than 55 h [15]. It could therefore be that the molecular mechanisms linking cell division and differentiation might be quite different in the two cell types, although the final result will be similar: cellular memory will show a high level of robustness in front of the cellular state change associated with the differentiation process.

Finally, it is tempting to speculate that the observed burst in entropy at the beginning of the differentiation sequence is helping the differentiating cells to overcome a memory process that is meant to prevent changes in cellular identity.

## Conclusions

To quantitatively investigate the interplay between transcriptional memory and gene expression variability during cell differentiation, we developed sophisticated experimental approaches to recover transcriptomes from related cells after one or two divisions while preserving lineage information at the single-cell level.

We found that the gene transcription profiles of sister cells exhibit more similarity to each other than to unrelated cells of the same type. More importantly, we observed greater disparities between differentiating sister cells compared to self-renewing sister cells. An increase in this divergence was evident from the first generation to the second generation when comparing differentiating cousin cells to self-renewing cousin cells.

These findings support the idea of a gradual erasure of transcriptional memory during the differentiation process. The initial increase in entropy observed in all systems examined to date during differentiation may facilitate cells in overcoming memory processes and allow the acquisition of a differentiated cell state.

### Supplementary Information

**Additional file 1: Figure S1.** [Technical validation of sister cell isolation method using CFSE intensity data and evaluation of background noise for self-renewing and differentiating cells]. **Figure S2.** [General structure of the data and characterization of the differentiation process]. **Figure S3.** [Histograms of cells viability]. **Figure S4.** [Euclidean distances comparison between generation 1 sister cells and non-related cells]. **Figure S5.** [Euclidean distances comparison between generation 2 sisters, cousins and non-related T2EC cells]. **Figure S6.** [Analysis of Manhattan distance sensitivity to gene set size]. **Table S7.** [Memory genes list with gene names and ENSEMBL gene ID].

**Additional file 2.** [CD34+ cells RT-qPCR raw data].

## Data Availability

Data tables are supplied as supplements files. scRNA-seq data are available in SRA repository under the BioProject accession PRJNA882056 at: https://www.ncbi.nlm.nih.gov/bioproject/?term=PRJNA882056 [58]. Previous data on mRNA half-life are available in the OSF repository at: https://osf.io/gkedt/ [46].

## Abbreviations

ACTB: Actin beta
BCAT1: Branched-chain amino acid transaminase 1
BH: Benjamini Hochberg
CFSE: Carboxyfluorescein diacetate succinimidyl ester
CTFR: Cell Trace Far Red
CTV: Cell Trace Violet
CTY: Cell Trace Yellow
EEF1A1: Eukaryotic translation elongation factor 1 alpha 1
ERCC: External RNA Controls Consortium
ERG: ETS transcription factor
FLT3: FMS-like tyrosine kinase 3
FTH1: Ferritin heavy chain 1
GAPDH: Glyceraldehyde 3-phosphate dehydrogenase
GATA1: GATA-binding factor 1
GRN: Gene regulatory network
H2AFZ: H2A family member Z
HBA1: Hemoglobin subunit alpha 1
HBAD: Hemoglobin subunit alpha D
HBBA: Hemoglobin subunit beta A
HK1: Hexokinase-1
HSC: Hematopoietic stem cells
h-SCF: Human stem cell factor
h-TPO: Human thrombopoietin
ID2: Inhibitor of DNA binding 2
IVT: In vitro transcription
KIT: Tyrosine protein kinase KIT (CD117)
LDHA: Lactate dehydrogenase A
PCA: Principal component analysis
PSI: Pounds per square inch
RHAG: RH-associated glycoprotein
RPL22L1: Ribosomal protein L22 like 1
RPS13: Ribosomal protein S13
scRNA-seq: Single-cell RNA sequencing
scRT-qPCR: Single-cell reverse transcription-quantitative polymerase chain reaction
SMC2: Structural maintenance of chromosomes 2
TAL1: TAL BHLH transcription factor 1
T2EC: TGF$\alpha$, TGF$\beta$ induced erythrocytic cells
TGF: Transforming growth factor
TMSB4X: Thymosin beta-4
UBA52: Ubiquitin A-52 residue ribosomal protein fusion product 1
UMAP: Uniform Manifold Approximation and Projection
UMI: Unique molecular identifier

## Code availability

R codes are available at: https://gitbio.ens-lyon.fr/LBMC/sbdm/sister-cells [59].
