# Supplementary material for: Differentiation is accompanied by a progressive loss in transcriptional memory
Source: BMC Biol. 2024 Mar 12;22:58. doi: 10.1186/s12915-024-01846-9 (PMC10929117; doi:10.1186/s12915-024-01846-9)
Supplement: Supplementary file 1 — Additional file 1: Figure S1. [Technical validation of sister cell isolation method using CFSE intensity data and evaluation of background noise for self-renewing and differentiating cells]. Figure S2. [General structure of the data and characterization of the differentiation process]. Figure S3. [Histograms of cells viability]. Figure S4. [Euclidean distances comparison between generation 1 sister cells and non-related cells]. Figure S5. [Euclidean distances comparison between generation 2 sisters, cousins and non-related T2EC cells]. Figure S6. [Analysis of Manhattan distance sensitivity to gene set size]. Table S7. [Memory genes list with gene names and ENSEMBL gene ID]. [file 12915_2024_1846_MOESM1_ESM.pdf]

Supplementary

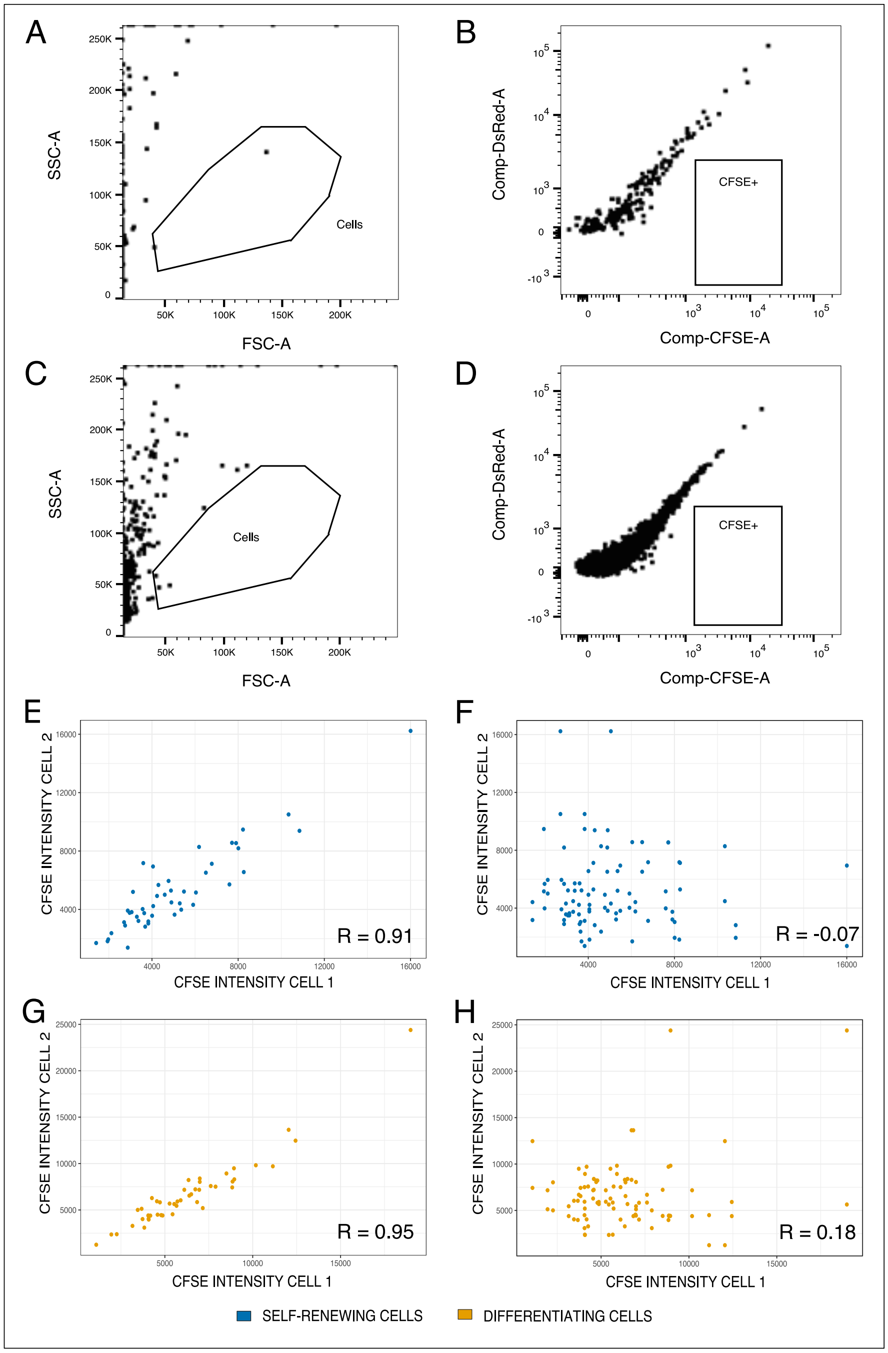

Figure S1: Technical validation of sister-cells isolation method using CFSE intensity data and evaluation of background noise for self-renewing and differentiating cells.

Figure S1: (A) Artefact detection in self-renewal medium. A few events are detected in the cell gate. (B) Artefact detection in self-renewal medium using CFSE signal (488nm, emission 530/30nm) versus auto-fluorescence (488nm, emission 585/42nm). No events is detected in the CFSE positive gate. (C) Artefact detection in differentiation medium. A few events are detected in the cell gate. (D) Artefact detection in differentiation medium using CFSE signal (488nm, emission 530/30nm) versus auto-fluorescence (488nm, emission 585/42nm). No events is detected in the CFSE positive gate. For graphs A to D all events are displayed. (E) CFSE intensity correlation between self-renewing sister-cells (Spearman R = 0.91). (F) CFSE intensity correlation of randomly paired self-renewing cells (Spearman R = -0.07). (G) CFSE intensity correlation between differentiating sister-cells (Spearman R = 0.95). (H) CFSE intensity correlation of randomly paired differentiating cells (Spearman R = 0.18).

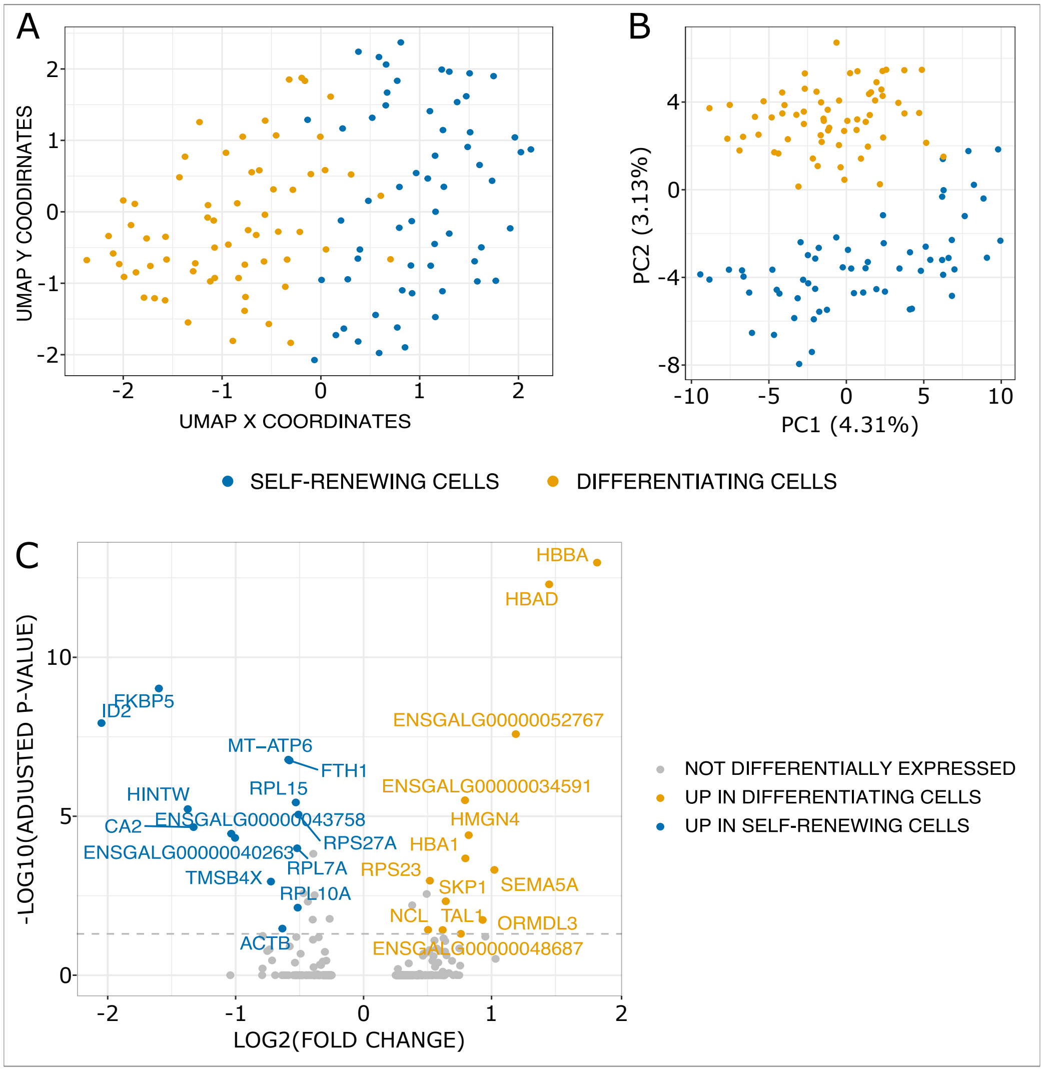

Figure S2: General structure of the data and characterization of the differentiation process.

Figure S2: Dimensional reduction and projection with UMAP (A) and PCA (B) of the scRNA-seq data on T2EC cells. Cells in self-renewal are in blue and differentiating cells are in yellow. (C) Volcano plot of genes differentially expressed between the two conditions. Genes are considered significantly differentially expressed when the fold change is equal or above 0,5 and adjusted p-value is below 0.05 (grey dotted line). Blue dots represent significantly up-regulated genes in self-renewing cells and yellow dots represent significantly up-regulated genes in differentiating cells.

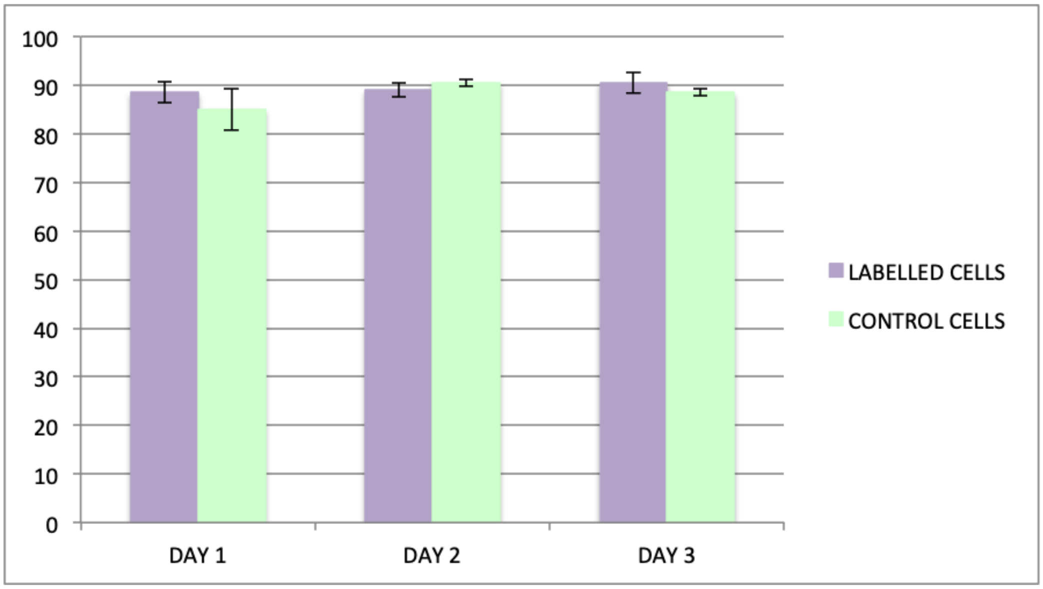

Figure S3: Histograms of cells viability.
Histograms of the percentage of viability, evaluated using Trypan Blue, during the cells staining (after day 1, 2 and 3) of the fluorescently labelled cells compared to negative control cells, on two biological replicates. T-test showed no significant differences.

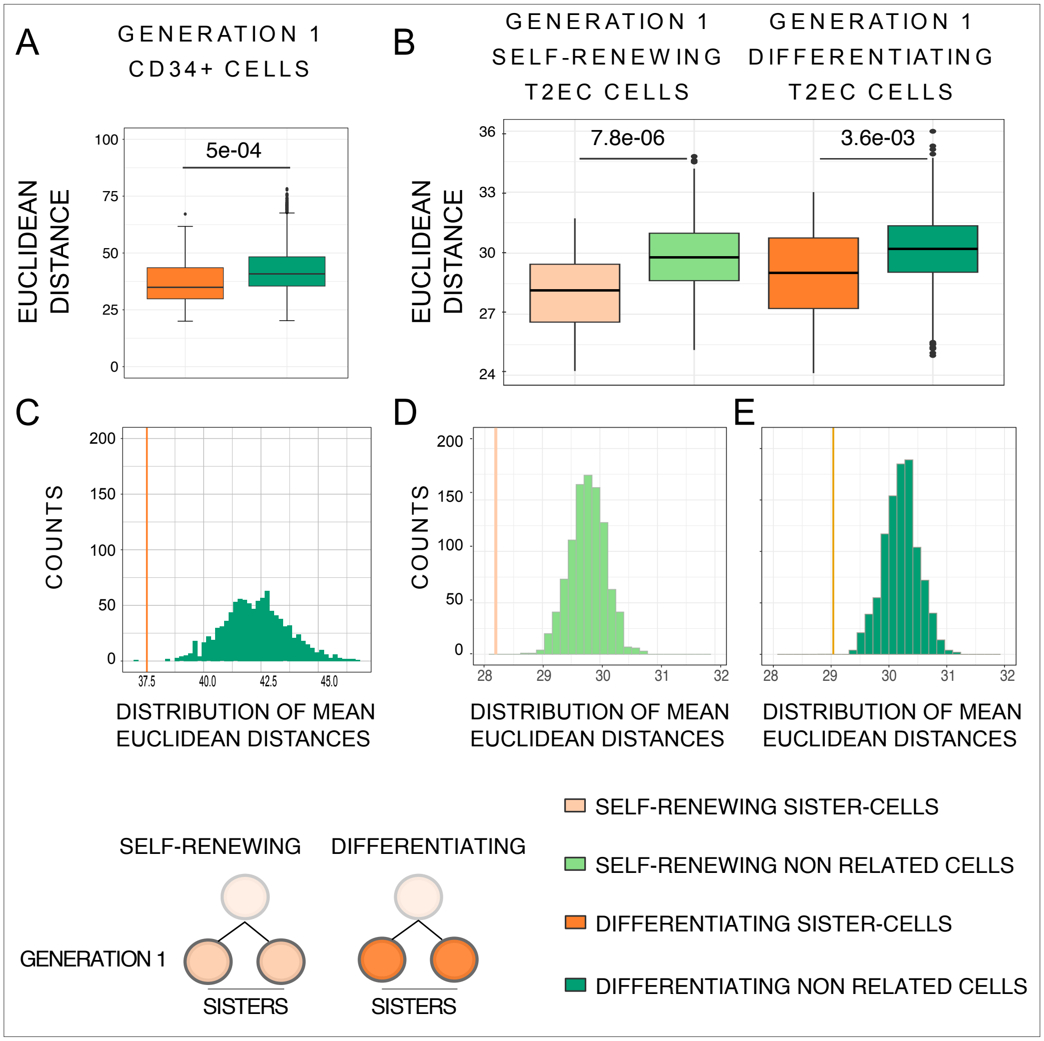

Figure S4: Euclidean distances comparison between generation 1 sister-cells and non related cells.

Figure S4: (A) Boxplot of Euclidean distances between the generation 1 CD34+ sister and non related cells. CD34+ sister-cells (43 couples) are in orange and CD34+ non related cells (3612 couples) in green. Euclidean distances were computed using all the 83 selected genes. Statistical comparison was performed using Wilcoxon test. (B) Boxplot of Euclidean distances between generation 1 T2EC sister and non related cells. Euclidean distances were computed between all cells from the same biological conditions using all the 1177 selected genes. Self-renewing sister-cells (30 couples) are in light orange and self-renewing non related cells (1740 couples) in light green, differentiating sister-cells (32 couples) are in orange and differentiating non related cells (1984 couples) in green. Statistical comparison was performed using Wilcoxon test. (C) Histograms of mean Euclidean distances of 1000 random draws of distances between 43 CD34+ non related cell pairs (green), compared to the mean distance between the 43 CD34+ generation 1 sister-cells pairs (orange line). (D) Histograms of mean Euclidean distances of 1000 random draws of distances between 30 T2EC self-renewing non related cell pairs (light green histogram), compare to the mean distance between the 30 T2EC self-renewing generation 1 sister-cells pairs (light orange line). (E) Histograms of mean Euclidean distances of 1000 random draws of distances between 32 T2EC differentiating non related cell pairs (green histogram), compare to the mean distance between the 32 T2EC differentiating generation 1 sister-cells pairs (orange line).

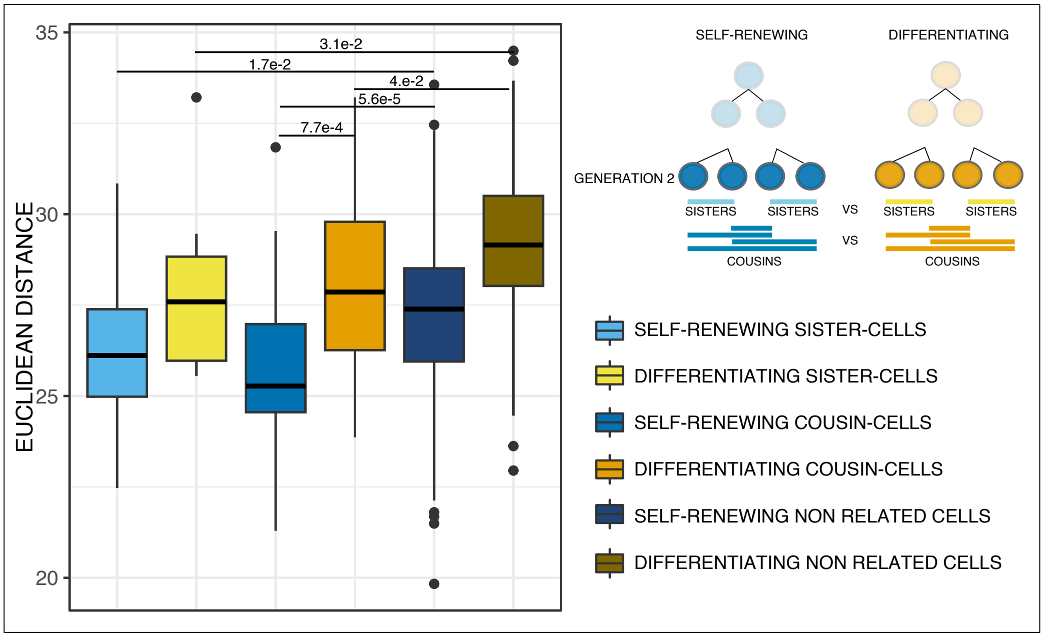

Figure S5: Euclidean distances comparison between generation 2 sisters, cousins and non related T2EC cells.

Boxplot of Enclidean distances between generation 2 sisters, cousins and non related T2EC cells. Euclidean distances were computed between all cells (32 self-renewing and 20 differentiating cells) from the same biological condition using the 983 selected genes. Self-renewing generation 2 sister-cells (16 pairs) are presented in light blue, self-renewing generation 2 cousin-cells (32 pairs) are in medium blue and self-renewing non related cells (448 pairs) are in dark blue. Differentiating generation 2 sister-cells (10 pairs) are in yellow, differentiating generation 2 cousin-cells (20 pairs) are in orange and differentiating non related cells (160 pairs) are in brown. Statistical comparisons were performed using Wilcoxon test.

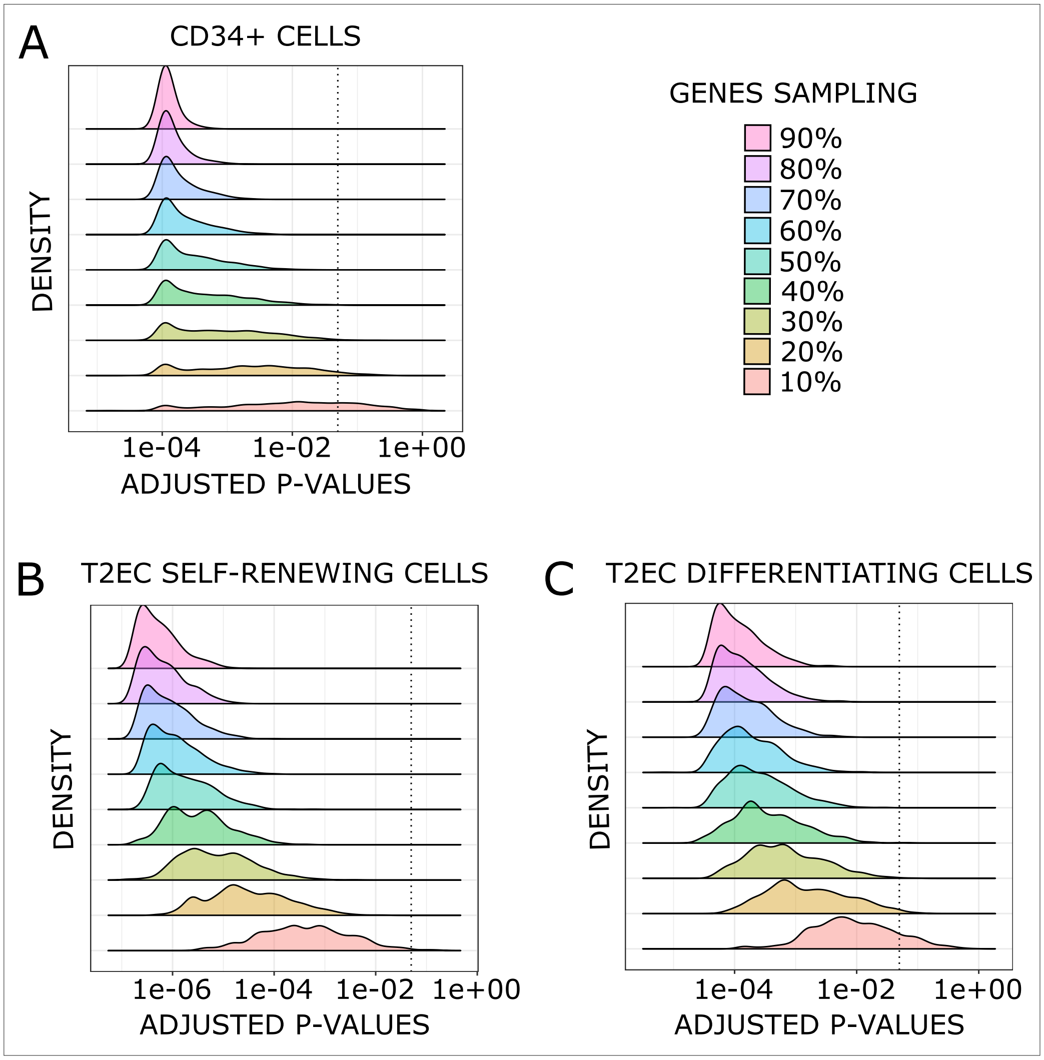

Figure S6: Analysis of Manhattan distance sensitivity to gene set size. Density plot illustrating the distribution of adjusted p-values resulting from the Wilcoxon tests conducted for mean Manhattan distance comparisons between sister and non related cells. This analysis was performed using varying percentages of randomly sub-sampled genes from the initial datasets, and the test was repeated 1000 times for each percentage. P-value of 0.05 is presented as dotted grey vertical line.

T2EC MEMORY GENES	
Gene_name	Ensembl_Gene_ID
ACTB	ENSGALG00000009621
ATP5G3	ENSGALG00000009286
ATP6V0C	ENSGALG00000009229
B2M	ENSGALG00000002160
CCNG1	ENSGALG00000001718
CD99	ENSGALG00000024488
CLTA	ENSGALG00000015326
DHRS7	ENSGALG00000011921
EEF1A1	ENSGALG00000015917
ENSGALG000	ENSGALG00000040263
ENSGALG000	ENSGALG00000043758
ENSGALG000	ENSGALG00000050548
ENSGALG000	ENSGALG00000052767
ENSGALG000	ENSGALG00000053077
ENSGALG000	ENSGALG00000053765
ESF1	ENSGALG00000034768
GAPDH	ENSGALG00000014442
H2AFZ	ENSGALG00000014023
HBA1	ENSGALG00000043234
HBAD	ENSGALG00000031597
HBBA	ENSGALG00000047152
HINTW	ENSGALG00000035998
HMGB2	ENSGALG00000010745
HSP90AA1	ENSGALG00000033212
ID2	ENSGALG00000035016
KPNA2	ENSGALG00000003584
LBR	ENSGALG00000009305
LDHA	ENSGALG00000006300
LY6E	ENSGALG00000041621
MLANA	ENSGALG00000019756
MRPS28	ENSGALG00000036749
MT-ATP6	ENSGALG00000041091
MT-COX3	ENSGALG00000035334
MT-ND2	ENSGALG00000043768
PLK1	ENSGALG00000006110
PPIA	ENSGALG00000028600
RHAG	ENSGALG00000016684
RPL13	ENSGALG00000006179
RPL22L1	ENSGALG00000009312
RPL37	ENSGALG00000014833
RPS23	ENSGALG00000015617
RTFDC1	ENSGALG00000007709
SAT1	ENSGALG00000016348
SEMA5A	ENSGALG00000028685
SH3BGRL3	ENSGALG00000038536
SMC2	ENSGALG00000015691
SOD1	ENSGALG00000015844
SPARC	ENSGALG00000004184
ST13P5	ENSGALG00000012007
TFRC	ENSGALG00000007485
TPD52	ENSGALG00000040167
TPX2	ENSGALG00000006267
TUBA1B	ENSGALG00000052192
UBA52	ENSGALG00000037716
UBE2I	ENSGALG00000006428

CD34+ MEMORY GENES	
Gene_name	Ensembl_Gene_ID
BCAT1	ENSG00000060982
GATA1	ENSG00000102145
HK1	ENSG00000156515
ACTB	ENSG00000075624
KIT	ENSG00000157404
CD38	ENSG00000004468
C22orf28	ENSG00000100220
ERG	ENSG00000157554
CD133	ENSG00000007062
CD74	ENSG00000019582

Table S7: Memory genes list with gene names and ENSEMBL gene ID. Memory genes were identified using linear models approach.
